# Bacterial family-VIII esterase displays dual activities: hydrolysis of polyester bioplastics and β-lactam antibiotics

**DOI:** 10.1093/ismejo/wrag203

**Published:** 2026-09-02

**Authors:** Harry Lerner, Diego Casaburi, Nele Charlott Meier, Léa Bernabeu, Marcel Eck, Stefan Mecking, David Schleheck

**Affiliations:** Department of Biology, University of Konstanz, 78457 Konstanz, Germany; Department of Biology, University of Konstanz, 78457 Konstanz, Germany; Department of Biology, University of Konstanz, 78457 Konstanz, Germany; Department of Chemistry, University of Konstanz, 78457 Konstanz, Germany; Department of Chemistry, University of Konstanz, 78457 Konstanz, Germany; Department of Chemistry, University of Konstanz, 78457 Konstanz, Germany; Department of Biology, University of Konstanz, 78457 Konstanz, Germany; Limnological Institute, University of Konstanz, 78457 Konstanz, Germany

**Keywords:** polyester biodegradation, plastic-degrading enzymes, β-lactamase, enzyme promiscuity, soil bacteria, polyester hydrolase, lipoprotein, family-VIII esterase

## Abstract

The plastisphere is a unique ecosystem with microbes colonizing and potentially degrading plastic debris in the environment, provided that the polymers are enzymatically accessible as substrates to drive microbial growth. It also harbors an unusually high occurrence of antibiotic resistance genes, suggesting plastic debris as a potential vector for antibiotic-resistant microorganisms. In this study, we investigated microbial communities in forest soil degrading an emerging type of bioplastics, aliphatic long-chain polyesters (LCAPs). Sequencing analysis revealed a family-VIII esterase strongly associated with LCAP depolymerization that showed high structural similarity to type C β-lactamases. Structural modeling and substrate docking analysis indicated catalytically favorable binding of both LCAP and β-lactam antibiotics. Furthermore, the active site appeared to be located in a large, wide-open groove, rather than in a tunnel, resulting in a protein with a striking “pac-man”-like structure. Heterologous expression and *in vitro* activity testing confirmed its dual functionality as plastic depolymerase and β-lactam hydrolase. Sequence analysis indicated the enzyme as membrane-associated lipoprotein likely to be directed to the outer membrane. The membrane anchoring of the enzyme may offer striking microbial-ecological benefits, by preventing enzyme loss especially in aqueous environments, by increased catalytic efficiency through high enzyme concentration at the cell–plastic interface, and by spatially linking catalysis with membrane transport, thereby limiting monomer loss to non-producing plastisphere-community members (cheaters). Hence, our study highlighted a plastic depolymerizing enzyme with a striking substrate spectrum, bridging plastics and antibiotics degradation, and provides intriguing perspectives for understanding the microbial physiology, ecology, and evolution of (bio)plastic degradation in the environment.

## Introduction

Plastic waste and its deterioration into micro- and nanoplastics, paired with slow biodegradation of most present-day plastic materials, has developed into a major environmental and human health concern [1–4]. In addition to being recalcitrant physical pollutants, plastic debris can act as a vector for transporting chemical contaminants and colonizing microbes [5–8]. Furthermore, microorganisms inhabiting the “plastisphere,” the biofilm community on plastic surfaces, have been shown to harbor disproportionately high levels of antibiotic resistance genes (ARGs), raising concerns about plastic-mediated dissemination of ARGs throughout ecosystems [9–13]. This enrichment of ARGs has been attributed to increased occurrence of horizontal gene transfer (HGT) within the plastisphere and to plastic-associated contaminants driving the selection for antibiotic resistance [10, 12]. Plastics are known to form “eco-coronas,” comprising layers of environmental (bio)molecules, including antibiotics, that adsorb to their hydrophobic surface [5–7]. The ARGs of concern involve, for instance, genes encoding for enzymes that inactivate antibiotics, such as β-lactamases, which are a subject of our present study. Here, an enzyme of the family-VIII esterases (carboxylic ester hydrolases; EC 3.1.1.-) with high homology to class C (serine-based) β-lactamases is concerned. These enzymes were initially considered incapable of β-lactam hydrolysis [14], but reports of β-lactamase activity have increased recently, including activity towards third- and fourth-generation cephalosporins, classified by the WHO as critically important antimicrobials [15–21].

The plastic materials themselves represent potential carbon and energy sources for microorganisms, provided that the polymer chains are biochemically accessible and that the microorganisms are capable of depolymerizing the materials outside of the cells into water-soluble monomers (or oligomers) that can be transported across the cell membrane and enter central metabolism. Despite hundreds of reported microbial plastic depolymerizing enzymes [22, 23], their diversity likely remains underrepresented, as many descriptions are based on sequence homology to few established benchmark plastic depolymerases, creating an “echo chamber” effect [24]. This is most evident for PETase-associated enzymes, a substantial fraction of which are variants or close homologues of the well-characterized *Ideonella sakaiensis* PETase [24]. Additionally, the ecological relevance of many such enzymes remains largely unconfirmed, as homology alone may not predict functionality and their prevailing characteristics often do not suggest specialization toward synthetic polymer degradation [25]. For instance, PETase homologues lack apparent substrate binding motifs [26, 27], such as the carbohydrate-binding modules of glycoside hydrolases or the substrate-binding domains of extracellular PHA depolymerases [28, 29].

Most plastic-degrading enzymes reported in the PlasticDB database are predicted to be freely secreted periplasmic or extracellular enzymes [22]. Although extracellular activity is a perquisite for degradation of insoluble, high-molecular weight polymers [30, 31], this may render host organisms vulnerable to the loss of both enzymes and hydrolysis products, which can diffuse away, particularly in aquatic environments [32]. In contrast, reports of membrane-associated plastic depolymerases that could degrade plastic directly at, or near the cell surface, appear disproportionally rare. For instance, out of 330 enzymes reported in the PlasticDB database (as of June 2026), only 3 are predicted to be transmembrane domain-containing enzymes and only 10 carry a canonical lipobox motif, which targets proteins to the Sec pathway and to signal peptidase II; subsequent lipidation of the invariant cysteine generates an N-acyl-diacylglycerol anchor that tethers the mature protein to the membrane [22] (see also Discussion).

In this study, we investigated the biodegradation of an emerging type of bioplastic, long-chain aliphatic polyesters (LCAPs), in a degradation field-trial in forest soil and in laboratory microcosms, also employing forest soil. LCAPs are chemically recyclable and biodegradable plastics comprised of ester-linked long-chain (e.g. C18 alkyl) dialcohol and dicarboxylic acid monomers (for example, polyester[1,18-octadecanediol-*alt*-1,18-octadecanedioic acid]; abbreviated PE-18,18) that can be biologically sourced from plant oils [33–35]. They can exhibit material properties similar to high-density polyethylene (HDPE) [33], making them a sustainable future alternative to conventional petrochemical polyolefins.

Here, we report on the identification of organisms and enzymes potentially involved in LCAP degradation, after performing metagenomic and amplicon-based sequencing analyses of the microbial communities in the microcosm set-up. We identified candidate enzymes with the predicted capacity to hydrolyze LCAPs, while matching the taxonomy of taxa enriched in the LCAP-treated microcosms. Among the top-candidate enzymes was a bacterial family-VIII esterase that showed similarities to both α/β-fold hydrolases and type C β-lactamases, and *in silico* substrate docking analysis suggested its potential for such dual functionality. This was confirmed through *in vitro* testing of the soluble enzyme, demonstrating the depolymerization of LCAP, as well as the hydrolysis of the β-lactam antibiotics. Furthermore, the enzyme was predicted to be an outer membrane-anchored lipoprotein, raising the possibility that it may be localized to the bacterial cell surface.

## Material and methods

Supplementary Materials and Methods provide further details on the methods summarized below.

### Polymer materials

Bioplastics poly(3-hydroxybutyrate-co-3-hydroxyvalerate) (PHBV), polycaprolactone (PCL), and cellulose were purchased and served as references. LCAPs (PE-2,18, PE-12,12, PE-18,18) and additive-free HDPE were synthesized in-house. Films of PE-18,18 and PE-12,12 were used for *in-situ* degradation assays, while milled PCL, PHBV, HDPE, and LCAP were employed in laboratory microcosms.

### *In-situ* degradation of LCAP films in forest soil and scanning electron microscopy

Film strips (≈1 × 3 cm) were mounted between grids and buried in humus-rich forest soil at the University of Konstanz Botanical Garden. Frames were covered with sieved humus and incubated *in situ* for 1 year. Retrieved films were air-dried, mounted on stubs, sputter-coated, and imaged using a Zeiss Auriga Cross Beam SEM at 5 kV with secondary electron detection.

### Microcosm degradation assays

Polymer biodegradation under controlled laboratory conditions was quantified by CO_2_ evolution from 1-g soil microcosms (triplicates) spiked with 50 mg powder of LCAPs, or of PHBV, PCL, HDPE, or cellulose as references. Moisture was adjusted to 70% of water-holding capacity, and samples were incubated at 30°C in sealed vials equipped with NaOH-filled CO_2_ traps. Trapped carbonate was periodically quantified (TOC-L analyzer, Shimadzu).

### DNA extraction and amplicon sequencing

Soil DNA (Day 164) was extracted using a CTAB-based protocol including PVPP cleanup. Bacterial and fungal communities were profiled via high-throughput sequencing of 16S rRNA gene (V3–V4 region) and ITS2 amplicons on the MiSeq System (Illumina). Sequence processing used the DADA2 pipeline, with taxonomic assignment via SILVA 138.1 and UNITE v9 databases. Community analyses and differential abundance testing (Log-fold change analysis, lfc) (ANCOMBC2) were conducted in R using phyloseq, vegan, and related packages.

### Metagenome sequencing and analysis

Triplicate DNA extracts were pooled by treatment and shotgun-sequenced (Illumina HiSeq). Reads were assembled (MEGAHIT v1.2.9) and processed in anvi’o (v7.1) for assembly, open reading frame prediction (Prodigal), and taxonomic classification (GTDB). Read mapping employed bowtie2. Contig taxonomy was assessed based on single-copy core gene (SCG) taxonomy inferred via hidden Markov models (HMMs) and/or gene-level taxonomic annotations, as implemented in the anvi’o workflow.

### Identification of plastic-degrading enzymes

Putative enzymes were identified by HMM searches against PlasticDB-derived models (HMMer v3.1b2, e-value <10^−3^), followed by BLASTP confirmation and SignalP-based secretion prediction. Candidate enrichment in LCAP-treated samples was evaluated using pre-sample normalized coverage and principal component analysis (PCA). High-confidence candidates were defined by stringent e-value and signal peptide criteria (see Supplementary Data).

### Structural modeling and docking

The LCPH1 enzyme structure was modeled using AlphaFold3. Docking of LCAP oligomer and β-lactam ligands was performed using AutoDock 4. Interactions were visualized and analyzed in ChimeraX. Multiple sequence alignments with homologous esterases were generated using MAFFT +DASH and visualized with Biopython.

### Heterologous expression and purification

The codon-optimized *lcph1* gene (without signal peptide) was cloned into pET-26b(+) and expressed in *Escherichia coli* Rosetta Gami 2 DE3. Cultures were induced with 0.4 mM IPTG at 18°C. Cells were lysed by ultrasonication, and the His-tagged protein purified by affinity chromatography (IMAC). Purity was verified by sodium dodecyl sulfate–polyacrylamide gel electrophoresis (SDS-PAGE) and concentration quantified fluorometrically.

### Enzyme assays

Esterase activity was assayed spectrophotometrically using *p*-nitrophenyl butyrate. Polyester depolymerase activity was determined from monomer release in reactions containing PCL or PE-2,18 powders and 2.5 μM enzyme, incubated at 30°C. Monomers were analyzed by HPLC-RID or HPLC-ESI-MS against authentic standards. Hydrolysis of β-lactam antibiotics was quantified by HPLC-DAD or HPLC-ESI-MS using gradient elution. IsPETase served as a reference enzyme for comparative activity tests.

## Results

### LCAPs show signs of *in-situ* degradation and are mineralized in forest-soil microcosms

**Figure 1:**
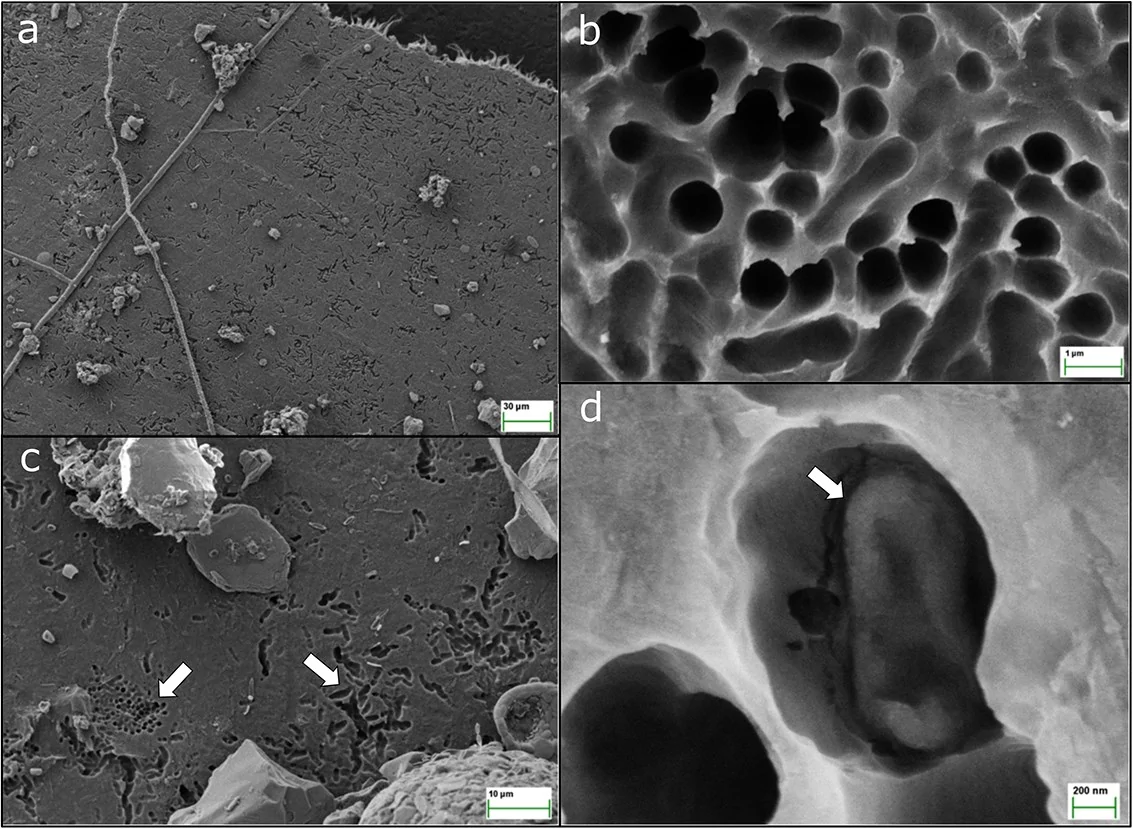
SEM images of LCAP films incubated undisturbed for more than a year in pristine forest soil. Representative images at increasing magnification are shown [see scale bars in panels (a–d)]. The structural damage on the bioplastic films after incubation, which were absent from non-incubated films (see Supplementary information, Fig. S2), obviously resulted from a very localized degradation of the plastic material, visible in form of microbial cell-sized cavities (a). The two arrows in panel (b) point at two different clusters of microbial cell-sized cavities with different shapes and sizes. A representative cluster of microbial cell-shaped cavities is shown in higher magnification in (c). The arrow in panel (d) points at an object inside of such a cavity that might represent a (damaged) microbial cell; however, these were found very rarely; empty cavities were more representative (a–c).

Films of LCAPs PE-12,12 and PE-18,18 were incubated in humus of a pristine mixed forest for nearly 15 months (for the setup, see Fig. S1) across the summers 2021 to 2022. The retrieved plastic films were macroscopically intact, but appeared more turbid and showed signs of breakage or embrittlement. Their examination by scanning electron microscopy (SEM) revealed substantial surface deterioration and material loss in form of frequent and clustered, approximately bacterial cell-sized, often rod-shaped cavities in the film surfaces (Fig. 1), which were absent from non-incubated films (Fig. S2). The observed surface alterations were attributed to initial microbial degradation of the plastic films, and the bacterial cell-shaped cavities prompted us to consider microbial cells that potentially degrade the polymers with extracellular depolymerase enzymes that are located, and potentially anchored, right at or very close to the cell surface (see below).

**Figure 2:**
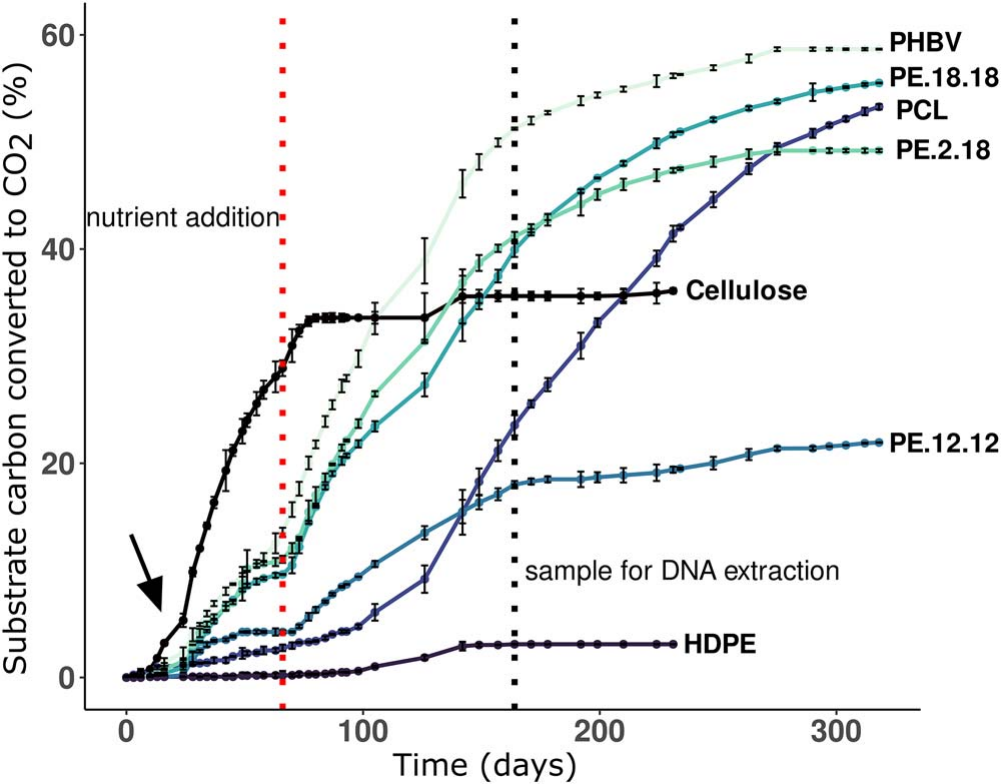
Substrate-specific respiration of forest-soil microcosms supplemented with powders of polymers PE-12,12, PE-18,18, and PE-18,2 in comparison to control polymers. Cumulative CO_2_ production is shown in percent of total polymer-carbon released as CO_2_, each after subtracting the basal CO_2_ production of untreated soil microcosms (blanks). The experiment was conducted using triplicate incubations for each polymer tested; standard deviation is indicated by error bars. The time point of external nutrient addition to the microcosms (see main text; N, P, and trace metals) is marked by a red dotted line (Day 66). The time point of sampling of all soil microcosms for total-DNA extraction and microbial community analysis by high-throughput sequencing is indicated by the black dotted line (Day 164). Control polymers used were PHBV, PCL, and HDPE, as well as cellulose. The arrow indicates the time point after which the capacity of the CO_2_ traps had been increased by using a higher NaOH concentration in the traps (see main text).

To assess LCAP degradation under more controlled conditions in the laboratory, and in parallel to the incubation of our field trial (see above), forest soil microcosms (triplicates) were set-up that were supplemented with milled LCAP (PE-2,18, PE-18,18, or PE-12,12), milled reference polymer (PHBV, PCL, HDPE) or cellulose powder. Substrate-induced CO_2_ evolution was monitored via NaOH-filled CO_2_ traps relative to non-spiked controls, for more than 300 days (Fig. 2).

During the first 23 days, insufficient capacity of the CO_2_ traps resulted in non-trapped CO_2_, reducing the accuracy of measurements during the initial incubation phase. The impact was likely highest for cellulose-spiked samples, where degradation started within few days (Fig. 2). The capacity of the CO_2_ traps was increased for the remainder of the experiment (see arrow in Fig. 2). Significant substrate-induced CO_2_ production was observed for all plastics except HDPE, where a maximum of only 3% substrate-carbon conversion to CO_2_ was recorded (Fig. 2). By Day 50, respiration plateaued, with cumulative CO_2_ production accounting for only ~10% of substrate-carbon for PHBV, PE-18,18, and PE 2,18, and <5% for PCL and PE-12,12 (Fig. 2). We considered that this likely reflected nutrient limitation, as indicated by the soil’s low pH (4.3), which may have affected nutrient bioavailability despite a favorable C:N ratio (Table S2). Accordingly, the microcosms were supplemented with a mineral-salts mixture (including ammonium, phosphate, and trace metals; see Supplementary Materials and Methods) at Day 66 (Fig. 2, red dashed line).

Upon nutrient addition, accelerated respiration was observed within few days, most pronounced for PHBV, PE-18,18, and PE-2,18, and to a lower extent also for PE-12,12 and cellulose (Fig. 2). In contrast, the CO_2_ production for HDPE and PCL exhibited no immediate increase, and background soil respiration was unaffected (Fig. S3). On Day 164, sub-samples for DNA extraction were retrieved (black dashed line in Fig. 2), and the incubation of the remaining soil was continued for up to 318 days.

After ~250 days, the cumulative CO_2_ production for PE-18,18 and PE-2,18, as well as for PHBV, plateaued between 49% and 58% of converted substrate carbon. These values match the idealized 50:50 carbon allocation observed during aerobic heterotrophic growth, where typically half of the available substrate carbon is respired for energy generation and released as CO_2_, and the other half is incorporated into the microbial biomass [36]. For PE-12,12, the cumulative CO_2_ production plateaued at only ~22%, indicating incomplete degradation. For PCL, mineralization reached ~53% by Day 318, indicating delayed but complete degradation. CO_2_ production for cellulose plateaued earliest after 77 days, reaching only ~36% of cellulose-carbon converted to CO_2_ (Fig. 2), likely due to non-trapped CO_2_ during the early incubation phase (see above).

### Phylogenetic analysis of bacterial and fungal communities in polymer-degrading soil microcosms

**Figure 3:**
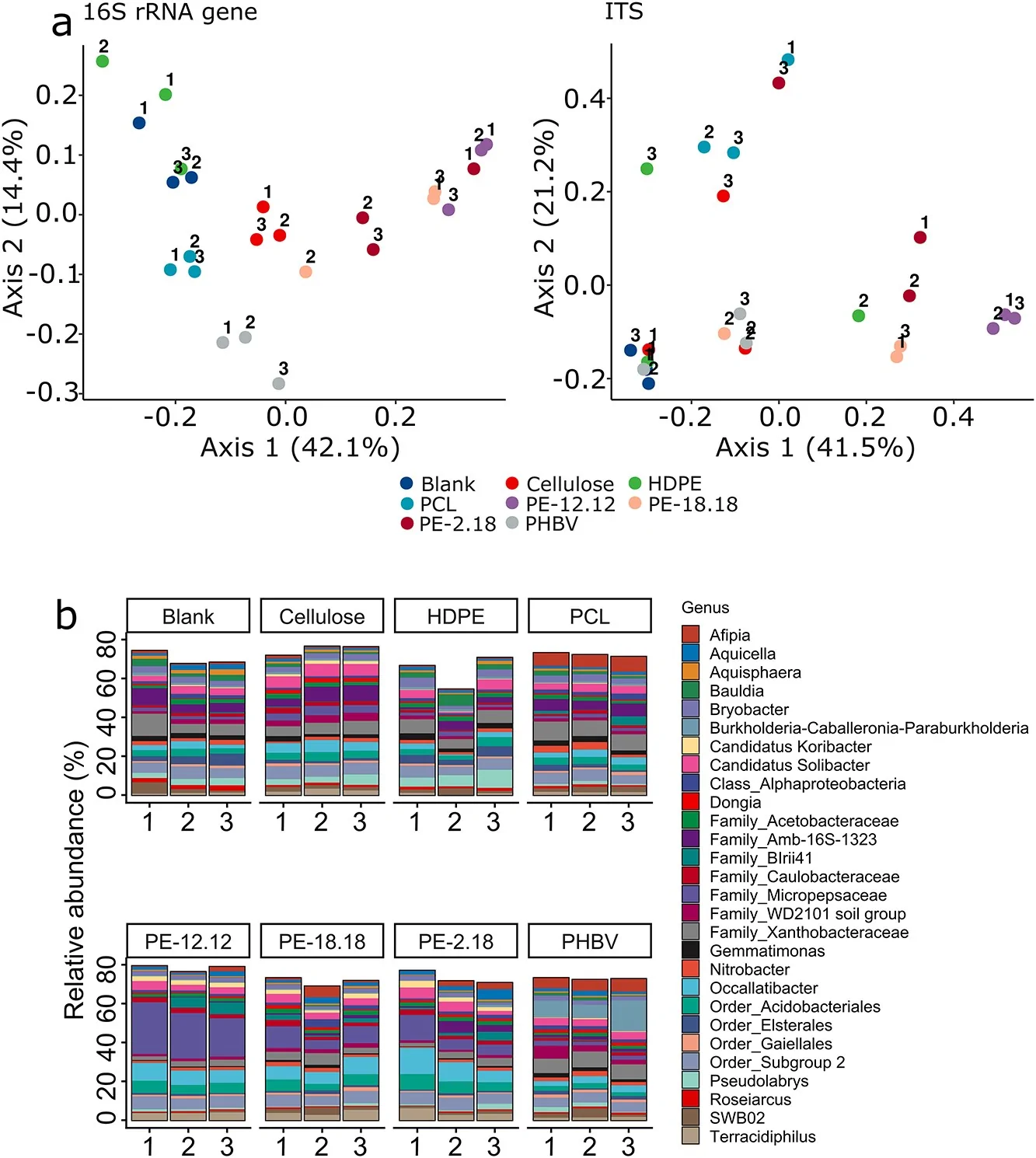
Principal coordinates analysis of 16S rRNA-gene and ITS amplicon sequencing data (a) and bar plots illustrating the 16S rRNA gene diversity (genus level) of the bacterial communities in soil samples incubated with the different polymers (b). The PCoA is based on Bray–Curtis dissimilarity metrics. Treatments are indicated in different colors, and individual replicates are numbered. Percentages given at the axes indicate the dissimilarity explained by the respective axis. (b) The community composition is shown for all replicate treatments. ASVs assigned to the same genus were combined. When a genus could not be determined with sufficient confidence, the last taxonomic level assigned is indicated. Only ASV groups with a mean relative abundance of >1% are depicted.

Substrate-specific changes in bacterial and fungal communities were assessed by amplicon sequencing of DNA isolated after 164 days. Principal coordinates analysis (PCoA) and PERMANOVA (*P* = .001) revealed clear grouping of bacterial and fungal communities corresponding to the respective treatments (Fig. 3a). The bacterial communities in the soil degrading PE-12,12, PE-18,18, and PE-2,18 were most dissimilar compared to non-spiked soil, and grouped closely together regardless of the LCAP variant used; only replicate 2 of the PE-18,18 treatment grouped closer to the cellulose group. The PHBV and cellulose replicates grouped close to the origin of the PCoA, and the PCL replicates grouped relatively close to the non-spiked controls. HDPE-treated soils also grouped near the non-spiked soil, although replicates were relatively dispersed on axis 2 (Fig. 3a). Similar grouping was observed for the ITS amplicon sequencing data, with LCAP-treated samples showing the highest degree of dissimilarity to non-spiked soil (Fig. 3a).

The LCAP-treated microcosms showed marked increases of ASVs assigned to family *Micropepsaceae*, as well as of closely related *Acidobacteriaceae* genera *Terracidiphilus* and *Occallatibacter*, whereas PHBV treatments showed a distinct increase of ASVs assigned to genera of the BCP group (*Burkholderia, Caballeronia*, and *Paraburkholderia*) (Fig. 3b). The genus *Afipia* appeared increased especially in PCL- and PHBV-treated microcosms, but also in four of the LCAP-treated replicates. The ITS-based fungal community showed a marked reduction of the GS11 clade compared to the non-spiked samples and an increased dominance of an ASV group with unidentified taxonomic affiliation (*incertae sedis*) in several LCAP-treated samples (Fig. S4a). Two PE-2,18 replicates showed an additional increase in *Rozellomycota* sequences, which likewise showed a strong increase in one cellulose replicate and two PCL replicates (Fig. S4a). Overall, the abundance changes in the fungal community appeared to be considerably lower compared to the observed changes in the bacterial communities, though few dominant groups with poor taxonomic resolution differed strongly between treatments and replicates (Fig. S4a).

Samples were further analyzed based on the log2-fold changes (LFC) in relative abundance between treatments and in comparison to the non-spiked and HDPE samples. For the ITS dataset, 10 ASVs showed significant abundance shifts in the LCAP group, all of which provided only poor taxonomic information (*incertae sedis*). For the PCL group, two ASVs of phylum *Rozellomycota* increased significantly (Fig. S4b).

**Figure 4:**
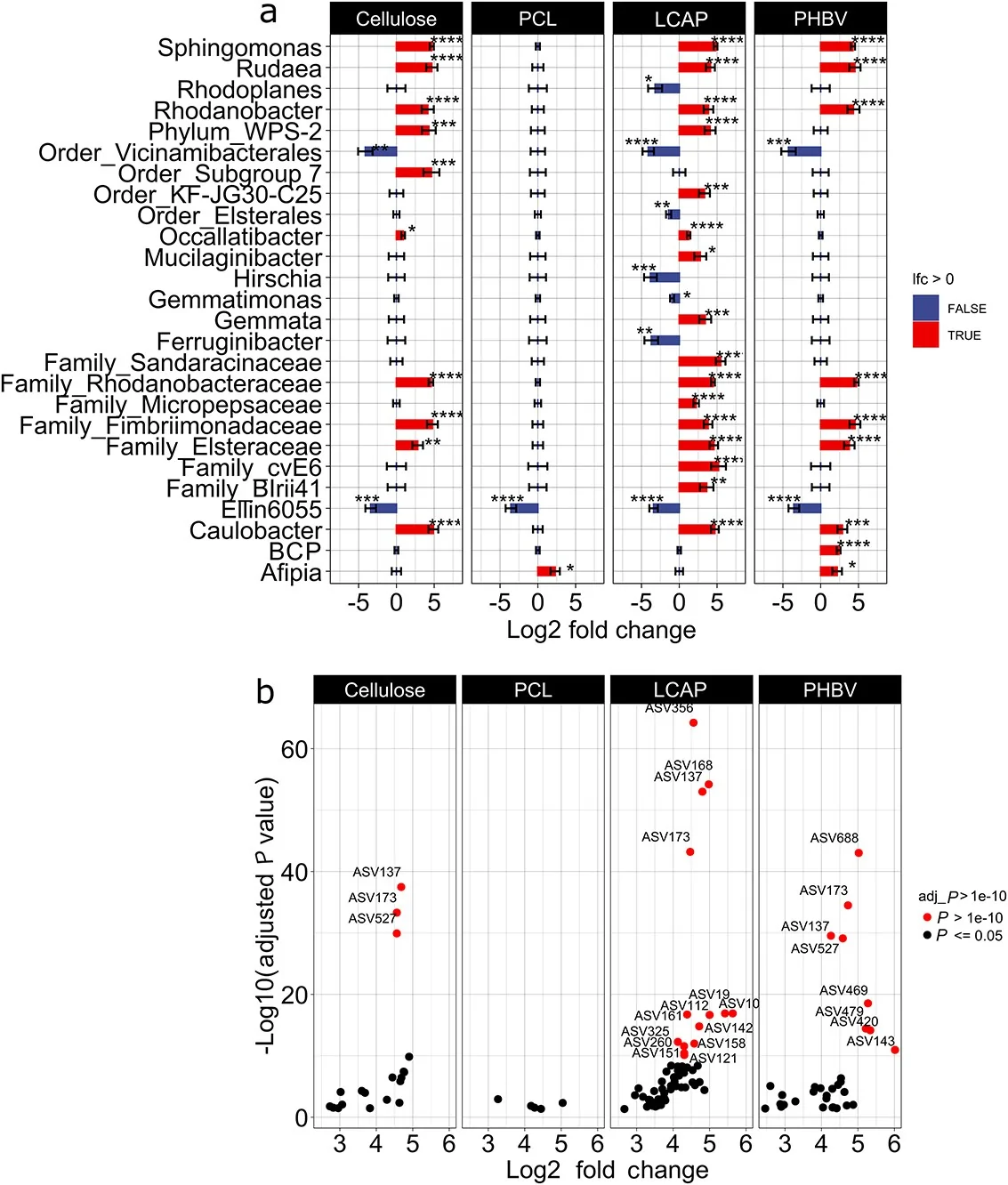
Log-fold change (LFC) analysis for differentially abundant bacterial taxa at the genus level (a) and ASV level (b) in the cellulose and PCL-, PHBV-, and LCAP-bioplastic degrading microcosms. Non-spiked microcosms (blanks) and the inactive HDPE-spiked microcosms were grouped together as reference. Likewise, all microcosms treated with the three types of LCAPs were grouped together, while replicates of the cellulose-, PCL-, and PHBV-treatments formed separate groups. To reduce the impact of sequencing artifacts and extremely rare ASVs, only ASVs with a count of ≥100 across 10% of samples were included in the analysis. Red bars (a) or dots (b) indicate a significant increase in a taxon’s abundance compared to the control condition. Blue bars indicate a reduction (a) and black dots (b) indicate taxa with an insufficient *P*-value. Taxa are displayed at the genus level (a) if classification was possible with sufficient confidence; otherwise, the highest reliable taxonomy level is indicated. Statistical significance of the LFC is indicated next to the bars (^*^P ≤ .05, ^**^P ≤ .01, ^***^P ≤ .001, ^****^P ≤ .0001). A table listing taxonomic affiliation and significance of each ASV shown in (b) can be found in the Supplementary information file (Table S3).

For the 16S rRNA-gene dataset, most differences were detected in the LCAP group, followed by the cellulose and the PHBV groups, whereas only sequences associated with *Afipia* were significantly increased in the PCL group (Fig. 4a). Seven taxa were uniquely increased in the LCAP group: the genera *Mucilaginibacter* (*Sphingobacteriia*) and *Gemmata* (*Planctomycetia*), the non-cultivated taxa KF-JG30-25 (*Acidobacteriia*), Blrii41 (*Acidobacteriia*), and cvE6 (*Verrucomicrobiae*), along with sequences assigned to the families *Sandaracinaceae* (*Myxococcia*) and *Micropepsaceae* (*Acidobacteriia*) (Fig. 4a). The genera *Sphingomonas, Caulobacter, Rudea, Rhodanobacter*, and further taxa belonging to the families *Rhodanobacteraceae, Fimbriimonadaceae*, and *Elsteraceae*, were significantly increased across the LCAP, as well as cellulose and PHBV groups.

At the ASV level and for the LCAP group, most significant increases (*P* < 10e^−10^) were observed for an ASV of the order *Acidobacteriales* (ASV356) and an ASV classified as *Pajaroellobacter* within the order *Polyangiales* (ASV168), which were exclusively observed for the LCAP group (Fig. 4b). Additional ASVs with highly significant increases, observed exclusively in LCAP-treated samples, belonged to the *Micropepsaceae* (ASV10, 19, 112, 158), *Terracidiphilus* (ASVs 161&325), *Oaccalatibacter* (ASV151) and *Elsteraceae* (ASV260). Highly significant increases were also observed for ASVs of *Sphingomonas* (ASV137), *Rhodobacteriaceae* (ASV173), and *Caulobacter* (ASV142); however, those were also increased for PHBV and/or for cellulose (Fig. 4b). ASVs that increased only for PHBV were exclusively related to the BCP group (ASV688, 479, 469, 420, 143), whereas an ASV related to *Tepidispherales* (ASV527) was also increased for cellulose (Fig. 4b).

### Metagenomics reveals candidate genes for putative extracellular LCAP depolymerase enzymes

**Figure 5:**
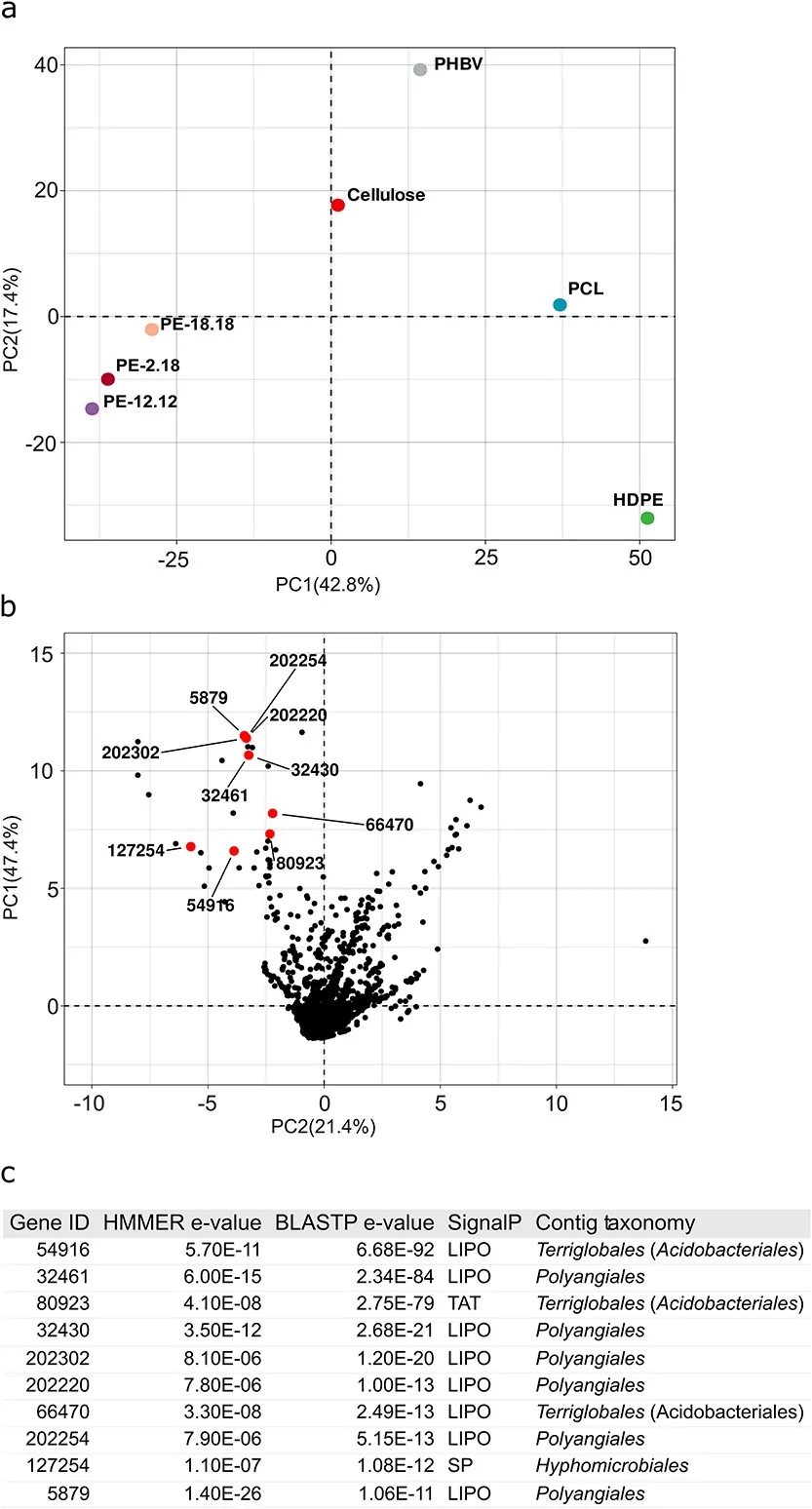
Principal component analysis. Shown are PCA results based on the per-sample normalized coverage values of contigs encoding putative depolymerases, comparing coverage patterns across treatments, and visualized (a) by microcosm treatment and (b) as volcano plot, and illustrating the clustering of individual proteins with the 10 highest scoring matches highlighted and labeled with their respective gene identifiers (Gene ID number). Percentages given at the axes indicate the dissimilarity explained by the respective axis. (c) Gene ID, HMMer, and protein Blast search e-values, along with predicted signal peptide and contig taxonomy of the top-matches highlighted in the volcano plot are shown in a tabular form.

Metagenome analysis identified in total 2624 unique candidate depolymerase genes based on HMM motifs of enzyme sequences stored in the PlasticDB [22]. PCA suggested a strong impact of polymer treatment on the coverage of contigs encoding these candidate plastic depolymerases (Fig. 5a). A PCA visualization revealed that out of the 2624 identified candidate enzymes, only a subset exhibited strongly differing contig coverage, highlighting their treatment-specific enrichment (Fig. 5b). All candidates with increased coverage for the LCAP treatment were manually curated and selected for further analysis (see Materials and methods).

Based on contig taxonomy, 4 of the top 10 best matches were taxonomically affiliated with the order *Terriglobales* (*Acidobacteriales* in the Silva taxonomy), 5 with the order *Polyangiales*, and 1 with the order *Hyphomicrobiales* (Fig. 5c). Out of these matches, four exhibited BLASTP e-values below 1e^−50^, suggesting a high sequence homology to previously described polymer-degrading enzymes in the PlasticDB database, although one was found to not correspond to a complete protein and was excluded from further analysis. This left three candidate enzymes, GeneIDs (GID) 32461, 54916, and 80923, as the three top-most striking candidates, since their taxonomic affiliation with the orders *Acidobacteriales* and *Polyangiaceae* closely matched the two most significantly increased ASVs in the LCAP group, ASV356 and ASV168, as indicated by the 16S rRNA-gene sequencing data (Fig. 4b).

All three LCAP-depolymerase candidates possess signal peptides for secretion, as predicted by SignalP-6.0 [37]. GID80923 is predicted to be secreted via the twin-arginine translocation (TAT) pathway, whereas GID32461 and GID54916 are predicted to be secreted via the general secretion (Sec) pathway. Additionally, GID32461 and GID54916 were found to contain a lipobox motif in the signal peptide, which targets the protein for lipid modification and anchoring to the cell membrane, rather than being secreted freely into the extracellular space [38].

Candidates GID32461 and GID80923 showed high sequence homology with PlasticDB entry 134 (36.5% and 38.8%, respectively) and likely belong to the carboxylesterase family (IPR002018). Their closest Uniprot matches were carboxylic ester hydrolases of *Sorangium cellulosum* (Uniprot ID A9FEC8) and *Alloacidobacterium dinghuense* (Uniprot ID A0A7G8BC64), respectively. In contrast, candidate GID54916 closely matched a β-lactamase of *Granulicella* sp. WH15 (Uniprot ID A0A7L5A8I1) and a metagenome-sourced esterase reported to depolymerize PLA (PlasticDB-entry 158) was the closest match in the PlasticDB database (42% identity, E-value 1.3e^−92^) [38]. Both GID54916 and PlasticDB-entry 158 possess a β-lactamase/transpeptidase-related domain (IPR012338). Due to these intriguing features, we decided to further characterize GID54916, both computationally and through heterologous expression and activity testing, as reported in the following.

### Modeling of the protein structure and substrate docking simulations for the predicted LCAP-depolymerase candidate GID54916

**Figure 6:**
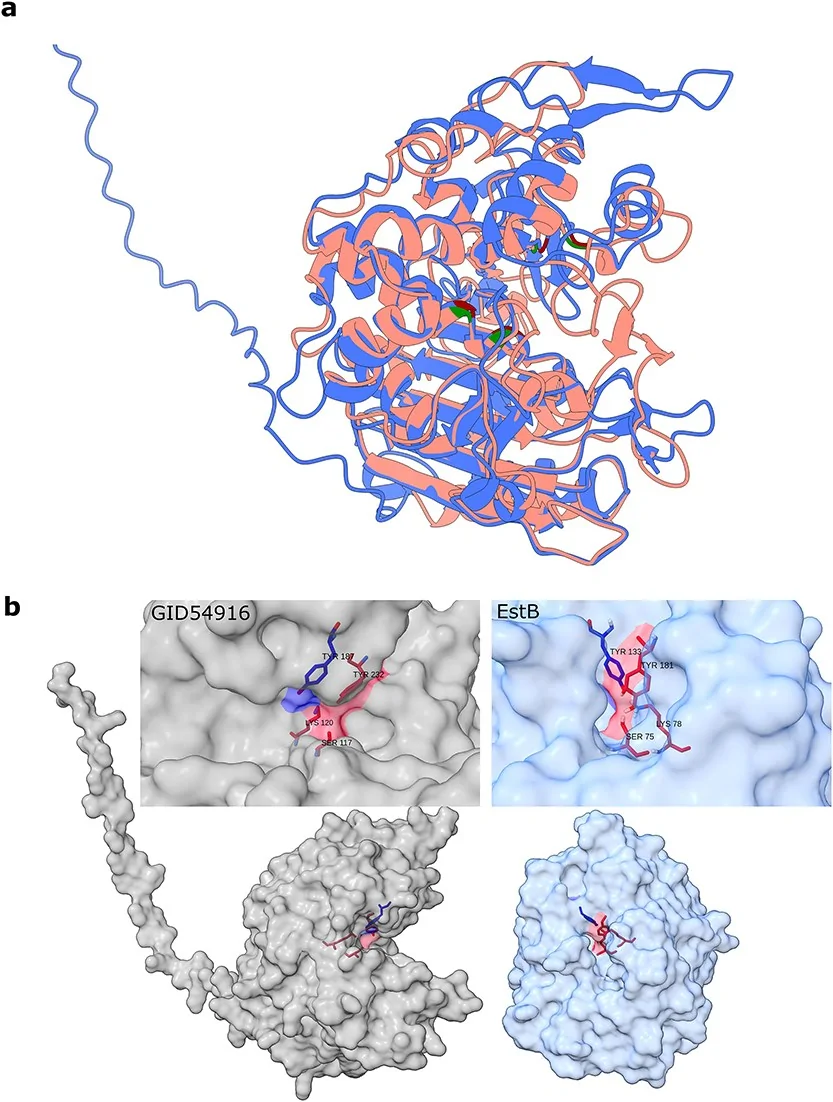
Predicted protein structures (a) and surface models (b) of GID54916 in comparison to its close homologue, characterized esterase EstB of *B. gladioli*. (a) Overlay of the two protein structures, with GID54916 in blue and EstB in pink. The conserved residues forming the active sites are marked in green for EstB and red for GID54916. (b) Comparison of the surface structures of GID54916 (light-gray) and of EstB (light-blue), illustrating a much more widely surface-exposed active site in a large accessible binding-groove as predicted for GID54916, rather than a tunnel as for EstB. The active-site surfaces are highlighted in red.

The AlphaFold 3-predicted structure of GID54916 revealed strong structural similarity to the esterase EstB of *Burkholderia gladioli*, a family-VIII esterase that shares several conserved features with class C β-lactamases (Fig. 6) [39]. Family-VIII esterases exhibit the esterase-atypical catalytic S-X-X-K motif, which matches the catalytic motif of class C β-lactamases [14, 39]. Other features shared between esterase-family-VIII enzymes and class C β-lactamases are the S-D-N loop and the K-T-G box that contribute to the shaping of the catalytic cavity [14, 39]. The S-X-X-K motif harbors the nucleophilic serine and the catalytic lysine, and the Y of the S-D-N loop is the third residue of the catalytic triad. The K-T-G box of class C β-lactamases corresponds to the W-X-G motif of family-VIII esterases (H-X-X for subfamily 3) [39]. A multiple sequence alignment of GID54916 and its closest PlasticDB matches, together with EstB and additional family-VIII esterases, as well as class C β-lactamases, confirmed that GID54916 has all the above mentioned conserved regions, grouping it within the type-VIII esterase family (Fig. S5).

**Figure 7:**
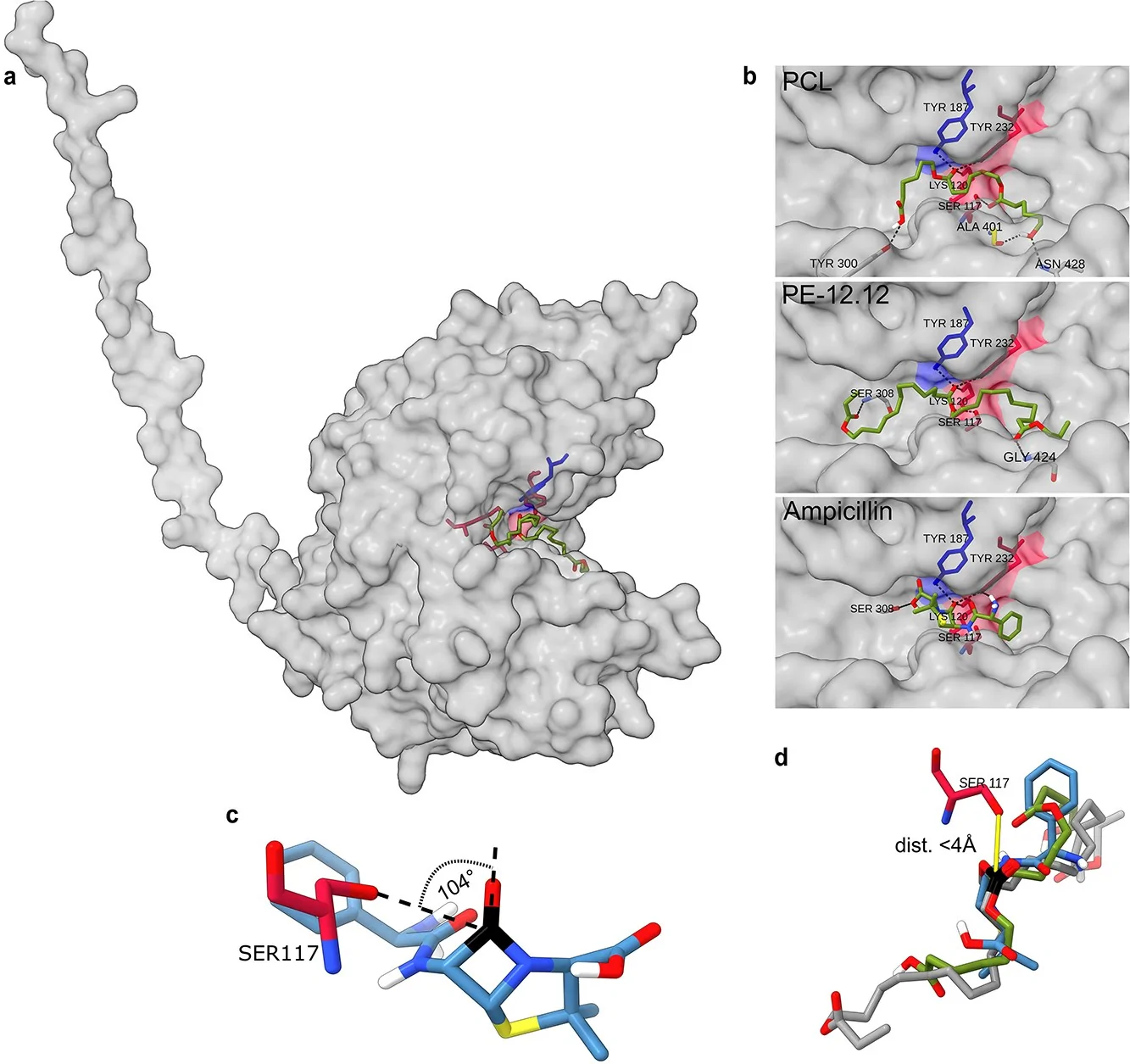
Alphafold-predicted surface structure of GID54916 with bound ligands. The catalytic triad residues (Ser117, Lys120, and Tyr232) are highlighted in red and Tyr187 in blue. (a) The PE-12,12 dimer bound to the enzyme surface is depicted in green with red sticks indicating the oxygen atoms of ester bonds. (b) Close-up of PCL, PE-12,12, and Ampicillin bound to the GID54916 active site. Potential hydrogen bonds formed between the ligands and catalytic enzyme residues are indicated by black dotted lines. (c) Visualization of the attack angle of Ser117 towards the carbonyl carbon of the Ampicillin β-lactam ring. (d) Overlay of the binding conformations of PE-12,12, Ampicillin and PCL with the carbonyl carbon of the esters (PE-12,12, PCL) or β-lactam group highlighted in black. The yellow line indicates the distance of the carbonyl carbon to the nucleophilic Ser117.

Although family-VIII esterases are known for their broad substrate spectrum and structural similarity to β-lactamases, these enzymes typically lack the ability to hydrolyze β-lactams, as is the case for EstB, due to the spatial limitations in its active site, which is constrained by a narrow tunnel-like structure that cannot accommodate the comparatively bulky β-lactam molecules [14, 39]. The structural model of candidate GID59416, in comparison, exhibited an active site that was much more exposed, forming a wide-open binding groove and resulting in a protein displaying a striking pac-man-like structure (Figs 6b and 7a).

Docking simulations using the polyester ligands PE-12,12 (dimer) and PCL (trimer), as well as the β-lactam antibiotic Ampicillin, resulted in docking solutions with similar binding energies, each placing the ligands in close proximity to the active site (Fig. 7). The docking solutions furthermore showed a favorable geometry for catalytic activity for all ligands. The carbonyl carbon of the ester or β-lactam group is in close proximity to the nucleophilic Ser117 (<4 Å), and the oxygen atom is in close enough proximity to Lys120, Tyr232, and Tyr187 to form hydrogen bonds, enabling the activation of the carbonyl carbon and the stabilization of the oxyanion during the catalytic process (Fig. 7b and d). Additionally, the attack angle for the β-lactam carbonyl likewise appeared to be suitable for nucleophilic attack, matching the reported range of the Bürgi–Dunitz angle of 100–110° (Fig. 7c) [40–42]. Hence, the structural modeling and docking simulations suggested that GID54916 might possess a broad substrate spectrum due to a widely accessible active site that may be able to accommodate and hydrolyze large substrates, such as β-lactam antibiotics and polyester chains.

### Heterologous expression of GID54916 and enzyme activity testing

To confirm the *in-silico* predicted depolymerization of LCAP and hydrolysis of β-lactams by candidate GID54916, a soluble variant of the protein (without secretion signal and lipobox motive) was heterologously expressed, purified and the quality of the preparations examined by SDS-PAGE (Fig. S6); esterase activity of the purified protein was confirmed using p-nitrophenyl butyrate as substrate in a colorimetric assay. Likewise attempted was the expression of GID54916 including its native secretion signal and lipobox motif in *E. coli*, in order to elucidate the cellular localization of the enzyme; however, these attempts produced no protein thus far, as evaluated by SDS-PAGE, most likely because of incompatibility of the signal sequence and/or lipobox motive of the *Acidobacteriales* GID54916 to the *E. coli* host; any further attempts will have to be reported in a future communication.

**Figure 8:**
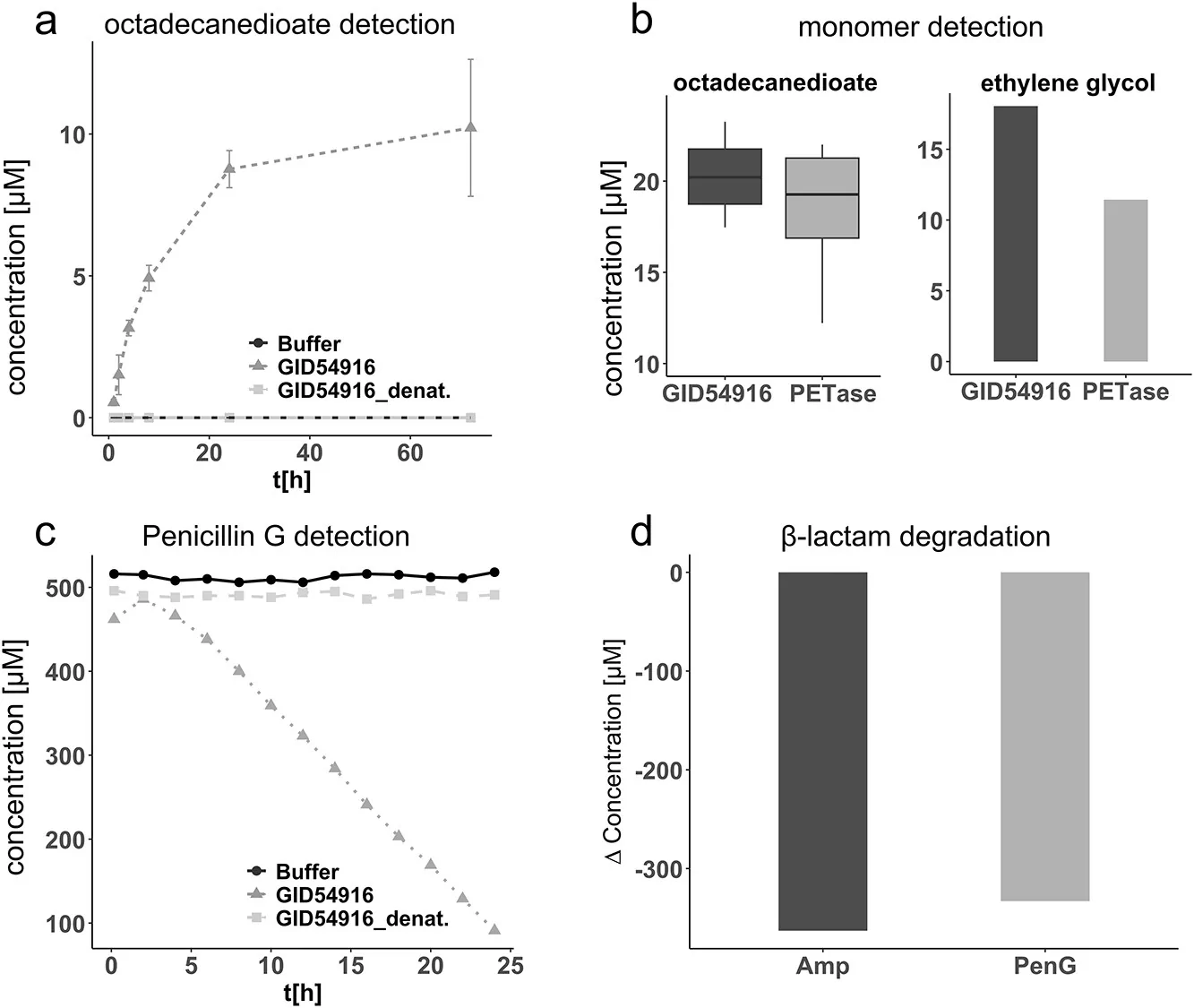
LCAP depolymerization and hydrolysis of β-lactam antibiotics by recombinantly produced GID54916 enzyme. (a) Time course of octadecanedioate monomer production in incubations of GID54916 with a powder of the LCAP PE-2,18 as substrate over 72 h, as compared to heat-denatured GID54916 and a buffer control. Reactions contained 5 mg of PE-2.18 powder and 2.5 μM of enzyme. The formation of octadecanedioate in the supernatant was analyzed by HPLC-ESI-MS in triplicate incubations; standard deviation is indicated by error bars. (b) End-point concentrations of both PE-2,18 monomers, octadecanedioate, and ethylene glycol, after incubation of PE-2.18 with GID54916, or with recombinantly produced *I. sakaiensis* PETase for comparison. Each 5 mg PE-2,18 and 2.5 μM enzyme were used. Octadecanedioate in the reactions (*n* = 3) was determined by HPLC-ESI-MS and ethylene glycol by HPLC-RID in a parallel reaction (*n* = 1), after 24 h and 18 h, respectively. (c) Time course of Penicillin G hydrolysis by GID54916 over 24 h, as determined by HPLC-DAD. The reaction contained 500 μM Penicillin G and 2.5 μM GID54916 and was analyzed every 2 h for 24 h. Control incubations with heat-denatured GID54916 or only buffer were included. (d) Concentration decreases of β-lactam antibiotics Penicillin G or Ampicillin after incubations with GID54916 for 24 h. Reactions contained 2.5 μM GID54916 and 500 μM Penicillin G or Ampicillin, and the antibiotics concentrations were determined before and after the incubations.

Enzyme assays with the truncated, soluble, and catalytically active version of GID54916 with the LCAP substrate PE-2,18 (powder) demonstrated octadecanedioate monomer release from the polymer over time, whereas incubations with heat-denatured or without enzyme showed no activity (Fig. 8a). The highest production was observed within the first 4 h, for which a specific activity of 0.5 U mg^−1^ was calculated. The other monomer of PE-2,18, ethylene glycol, was also released in comparable proportions, as followed by end-point determination in parallel reactions (Fig. 8b). Reactions with recombinant *I. sakaiensis* PETase and PE-2,18 produced similar amounts of monomers (Fig. 8b). GID54916 also depolymerized PCL, however at significantly higher activity (Fig. S7; up to approx. 2 mM caprolactone in 24 h), likely owing to a higher frequency of ester bonds and a low molecular weight and/or crystallinity of PCL, compared to PE-2,18.

The β-lactam hydrolysis was demonstrated by Penicillin G disappearance in incubations with active GID54916, whereas incubations with heat-denatured or without enzyme showed no activity (Fig. 8c and Fig. S8). Penicillin G disappeared nearly completely within 24 h; a specific activity of 8.92 U mg^−1^ was calculated (Fig. 8c and Fig. S8). Ampicillin also disappeared, as shown by end-point determination in parallel reactions (Fig. 8d). Production of the corresponding intramolecular hydrolysis products penicilloic acid and ampicilloic acid, respectively, by GID54916 was confirmed using LC-ESI-MS analysis (Fig. S9), in comparison to PETase, which did not convert Penicillin G and Ampicillin (Fig. S10). Finally, inactivation of Ampicillin by GID54916 was furthermore confirmed in a bio-assay, by loss of the inhibitory effect on growth of *E. coli* K12, using disc diffusion assays in which the enzyme was added to Ampicillin-containing filter-paper discs on agar plates, resulting in loss of the antibiotic effect (Fig. S11).

## Discussion

This study was initiated to investigate the biodegradation of an emerging type of bioplastics by environmental microbial communities, here, of forest soil, and has led to the discovery of a bacterial, most likely membrane-anchored family-VIII esterase (GID54916, termed Long-Chain Polyester Hydrolase 1, LCPH1) that catalyzes polyester, as well as β-lactam hydrolysis, in a rare dual functionality. Our results are contributing not only important information on microbial LCAP degradation relative to reference (bio)plastics, and on likely involved phylotypes in forest soil, but are also providing new and intriguing future perspectives for understanding the microbial physiology, ecology, and evolution of (bio)plastic degradation in the environment, as is discussed in the following.

The microcosm experiments (Fig. 2) demonstrated near-complete mineralization of the LCAPs PE-2,18 and PE-18,18 within 8–10 months, at rates similar to those observed for bioplastics PHBV and PCL. In contrast, PE-12,12 degradation reached a plateau at ~20% of polymer-carbon conversion to CO_2_, despite comparable crystallinity and polymer-chain lengths (molecular weight) of the LCAPs tested. This result confirms the previously demonstrated biodegradability of LCAPs using agricultural soil under mesophilic conditions and compost under thermophilic conditions [34, 35, 43]. The varying degradation kinetics observed across studies are likely resulting from differences in microbial inoculum, incubation conditions, and/or LCAP preparations, which clearly warrants further investigation.

The microcosm experiments furthermore highlighted that also nutrient limitations can restrict degradation of plastics. Supplementing N, P, S, and trace metals to the forest soil markedly improved degradation rates (Fig. 2), suggesting that natural environments might not exhibit their full plastic degradation capacity due to nutrient deficiencies, as far as well-degradable (bio)plastics are concerned. This is supported by studies showing a positive effect of nitrogen-fixing bacteria on polymer degradation [44], as well as improved degradation when additional N was supplied in the polymer matrix or through the inclusion of natural N-containing fillers [45, 46]. However, the most profound hindrance of efficient microbial degradation of plastics was deemed to be insufficient water availability, as concluded from our field trial with LCAP films (Fig. 1) incubated in undisturbed, unusually dry forest soil (i.e. during very dry years 2021 and 2022 in Germany), for about as long as the LCAP microcosms.

Across microcosm samples, 2624 coding genes were identified with significant similarity to HMM motifs obtained from reported plastic depolymerases. PCA based on normalized contig coverage enabled prioritization of candidate genes enriched in LCAP-treated samples (Fig. 5). Nine of the top 10 candidates showed contig-level affiliation with *Acidobacteriales* or *Polyangiales*, hence, consistent with the amplicon-based abundance profiles (Figs 3 and 4).

Among these, GID54916 (*Acidobacteriales*) encoding LCPH1 was selected for further analysis due to its prediction as a secreted lipoprotein with structural similarity to type C β-lactamases. In gram-negative bacteria, known lipoproteins are typically anchored to the inner leaflet of the outer membrane [47], although recently improved understanding of gram-negative lipoproteins suggests that both cell-surface exposures, including anchoring to the outer leaflet of the outer membrane, as well as further secretion of periplasm-facing lipoproteins via outer membrane vesicles (OMVs), may be more prevalent than previously estimated [48–53].

This finding captured our attention, since membrane association of known plastic depolymerases appears exceptionally rare. To the best of our knowledge, the only directly reported lipoproteins involved in plastic degradation are the *I. sakaiensis* MHETase, which, however, degrades the water-soluble PET dimer MHET (mono-2-hydroxyethyl terephthalic acid) [54], and oxidized-PVA hydrolase (OPH) of *Pseudomonas* sp. strain VM15C, which catalyzes the degradation of pre-oxidized polyvinyl alcohol [55]. Like the MHETase enzyme, OPH was suggested to act in the periplasm [56]. Although there appear to be no explicit reports of extracellular plastic-depolymerizing lipoproteins to date, these attributes might have been overlooked, or considered irrelevant. For instance, the membrane-associated polyurethane (PU) esterase (PudA; PlasticDB entry 13) contains a lipobox motif, but was not reported as a lipoprotein [57]. In total, 10 lipobox motif-containing enzymes were identified in the PlasticDB database; however, this feature is only directly addressed in case of MHETase and OPH. Apart from lipoproteins, other membrane-associated plastic-degrading proteins are likewise scarcely reported, such as the PET-degrading transmembrane esterase in gram-positive *Rhodococcus pyridinivorans* P23 [58]. Furthermore, the involvement of OMVs in the degradation of PU oligomers was demonstrated, which implies another membrane-associated secretion mechanism involved in plastic degradation [59].

These observations suggest that depolymerases anchored to the outer cell surface (ectoenzymes) may be a yet underrecognized strategy in plastic-degrading natural microbial communities. This strategy is well described for plant polymer-degrading bacteria, which employ cellulosomes associated to the cell surface [60], as well as OMVs for plant-cell wall degradation [61–63]. Considering that depolymerizing enzymes for natural and synthetic polymers often belong to the same enzyme families, the adaptation and evolution for improved degradation of synthetic man-made polymers appears likely. Such a functional display of the depolymerases attached to the outer cell surface—coupled to the surface-attached biofilm lifestyle of extracellular plastic-degrading bacteria in the environment (in the plastisphere)—implies several advantages, by (i) preventing loss of enzymes especially in aqueous environments, (ii) increasing catalytic efficiency through high local enzyme concentration directly at the bacterial cell–plastic interface, and (iii) through spatially coupling monomer formation and the uptake of monomers into the cells, thus limiting losses of monomers to non-producing plastisphere community members (“cheaters”) [32]. Indeed, such advantages were demonstrated in a biotechnological setting, showing that a PETase functionally displayed on the surface and combined with hydrophobin-mediated cellular plastic adhesion in genetically modified yeast cells, resulted in an up to 329-fold increased efficiency compared to freely dissolved PETase [26]. Such a functional display of the depolymerases on the cell surface, or close to the cell surface when secreted via OMVs, might also have resulted in the single-cell-shaped voids (“sinkholes”) in the plastic films as retrieved from the forest soil after our field trial (Figs 1 and S2).

Even though an experimental confirmation of the cellular localization of LCPH1 must await future research, the encoded secretion and lipobox motives, as well as the absence of an Asp residue at the +2 position, which would act as strong sorting signal for lipoprotein-anchoring to the inner membrane [64], already strongly supports an outer-membrane association of LCPH1. The genomic context of the LCPH1-encoding contig provides further support, in addition to suggesting potential involvement of OMVs in its secretion: in addition to LCPH1, the contig includes genes for LolC/LolE-like permeases that traffic lipoproteins to the outer membrane [64], a cell-wall-remodeling enzyme MepM/NlpD (murein endopeptidase) implicated in envelope dynamics and OMV biogenesis [65] and a GGDEF-domain protein, indicating involvement of c-di-GMP signaling as key regulator of biofilm formation, surface attachment, and coordinated secretion responses, including OMV formation [66, 67]. Additionally, directly adjacent to the *lcph1* gene, the contig encodes a TonB-dependent receptor for potential uptake of complex degradation products, as previously suggested for nutrient acquisition in conjunction with surface-exposed substrate-binding lipoproteins in *Bacteroides fragilis* [48, 68]. TonB-dependent receptors were also found to be enriched in OMVs [62, 69]. Together, the contig-level co-occurrence of these features strongly support a model in which LCPH1 is surface-exposed, and may be secreted via OMVs, along with synergistic enzymes, during colonization of plant material—or other polymeric substrates such as LCAPs.

The predicted structural features of LCPH1 also highlight intriguing aspects. LCPH1 showed striking similarities to family-VIII esterases, and particularly EstB from *B. gladioli*, leading to our assignment of LCPH1 to this enzyme family (Fig. S5). Other than EstB, however, LCPH1 revealed a pac-man-like shape with an intriguingly wide, surface-exposed active site with the potential to bind large substrates (Figs 6b and 7a). This was confirmed via substrate docking simulations, which showed the favorable binding of polyester chains of PCL and PE-12,12, as well as of Ampicillin (Fig. 7). These computational predictions were confirmed experimentally, demonstrating both the LCAP depolymerase and β-lactamase activity of the enzyme (Fig. 8). Although reports of β-lactamase activity among family-VIII esterases keep increasing [15–21], there have been no prior reports of a family-VIII esterase exhibiting both plastic depolymerase and β-lactamase activity [38, 70, 71]. This overlap in substrate spectrum was also recently reported for a metagenome-derived esterase PET40, which exhibited both PET depolymerase and β-lactamase activity, despite lacking the canonical β-lactamase fold [72].

This dual activity of LCPH1, and of PET40, poses interesting questions regarding the ecological roles of these enzymes and potential unexpected consequences of plastic waste in the environment. Based on our results, it is likely that LCPH1 provides resistance to β-lactam antibiotics, while also conveying additional fitness by enabling the utilization of (synthetic) polyester substrates. An outer-membrane anchoring of LCPH1 seems favorable also for its β-lactamase functionality, enabling extracellular inactivation of antibiotics before they reach their target, a mechanism previously described in several cases for β-lactamases, such as outer-membrane anchored OXA-type carbapenemases of *Acinetobacter* spp. (also via a lipobox motive) [73]. It is tempting to speculate whether this duality might therefore result in a co-selection of antibiotic-resistant microbiota in the plastisphere as a result of (polyester) plastic exposure, contributing to the frequently reported high incidence of ARGs in the plastisphere [9–13]. Furthermore, it is tempting to speculate that, on the other hand, such enzymes, initially recruited through co-selection processes, could evolve towards specialization in synthetic polyester degradation under appropriate selective pressures. From an annotation-based perspective, this raises the possibility that sequences recovered from plastisphere environments may be incorrectly classified as ARGs, while primarily functioning in plastic degradation, and conversely, enzymes such as LCPH1 and PET40 may be annotated for their polyester hydrolase feature despite exhibiting the dual functionality in their hosts.

## Supplementary Material

Family-VIII_esterase_Lerner_et_al_SI-file_wrag203

## Data Availability

The metagenomic and amplicon sequencing raw data have been deposited at EBI with the identifier PRJEB96049.
