# Supplementary material for: Bacterial family-VIII esterase displays dual activities: hydrolysis of polyester bioplastics and β-lactam antibiotics
Source: ISME J. 2026 Sep 2;20(1):wrag203. doi: 10.1093/ismejo/wrag203 (PMC13533575; doi:10.1093/ismejo/wrag203)
Supplement: Supplementary file 1 [file family-viii_esterase_lerner_et_al_si-file_wrag203.pdf]

Bacterial Family-VIII Esterase with Dual Activities: Hydrolysis of Bioplastics and of β-Lactam Antibiotics

Harry Lerner, Diego Casaburi, Nele Charlott Meier, Léa Bernabeu, Marcel Eck, Stefan Mecking and David Schleheck

University of Konstanz, Germany

Supplementary Information

Contents

A. Supplementary Materials and Methods

B. Supplementary Figures

Figure S1. Photographs illustrating the placement of the LCAP films mounted into 'HYDRA test frames' in forest soil.

Figure S2. Scanning electron microscopy (SEM) images of PE-12,12 and PE-18,18 films incubated in pristine forest soil for about one year.

Figure S3. Average daily CO_2 rates in soil blank microcosms.

Figure S4. Bar plots illustrating the ITS gene diversity (genus level) of the fungal communities in soil samples incubated with the different polymers and Log-fold change (LFC) analysis for differentially abundant taxa.

Figure S5. Multiple sequence alignment of LCPH1 and other family-VIII esterases.

Figure S6. SDS-PAGE analysis of the heterologous expression and purification of His-tagged LCPH1.

Figure S7. Discontinuous enzyme assay demonstrating ε-caprolactone monomer release from PCL plastic by recombinantly produced GID54916.

Figure S8. Discontinuous enzyme assay demonstrating the disappearance of Penicillin G by LCPH1.

Figure S9. Confirmation by HPLC-ESI-MS of hydrolysis of Ampicillin and Penicillin G by LCPH1.

Figure S10. Confirmation by HPLC-ESI-MS that PETase cannot hydrolyse Ampicillin or Penicillin G.

Figure S11. Ampicillin disc diffusion assay with and without LCPH1 exposure.

C. Supplementary Tables

Table S1. Analysis of the utilized forest soil according to Albrecht Standard.

Table S2. Concentrations of trace metals added to the soil microcosms.

Table S3. Taxonomic affiliation of differentially abundant ASVs.

D. Supplementary References

A. Supplementary Materials and Methods

Polymer materials. Poly(3-hydroxybutyrate-co-3-hydroxyvalerate) (PHBV) was purchased from Natureplast (grade: PHI002, Mondeville, France). Polycaprolacton (PCL) was purchased from Sigma-Aldrich (Product ID: 440752, St. Louis, MO, United States) as well as cellulose powder (Sigmacell Type 20). Long chain aliphatic polyester (LCAP) bioplastics PE-2,18, PE-12,12, and PE-18,18 were synthesized in house as previously reported [1]. The numbering indicates the chain length, i.e., the number of carbon atoms in the diol (first number) and dicarboxylate (second number) monomeric units. High density polyethylene (HDPE) was likewise synthesized in house to avoid potential contamination with industry-typical additives, using ethylene, 5 mL of toluene as solvent and 5 μmol of naphtyl-MeOBIPh-Ph as a catalyst.

For the degradation test in forest soil, plastic films of PE-18,18 and PE-12,12 material were used, produced as previously described [1]. For the degradation test with forest soil incubated in laboratory microcosms, plastic materials PHBV, HDPE, PE-2,18, PE-12,12, and PE-18,18 were used, each milled to a powder after immersion of samples in liquid nitrogen using a Retsch ZM 200 Ultra Centrifugal mill (Retsch GmbH, Haan, Germany) at 14,000 rpm, equipped with a ring sieve with a mesh size of 1 mm.

***In situ* degradation assay.** Film strips of approx. 1 cm x 3 cm in size were mounted in between plastic grids in frames [2]. For deployment of the materials into forest soil at the Botanical Garden of the University of Konstanz (mixed forest, predominantly beech and pine; coordinates, 47.691552, 9.179949), the top layer of litter was carefully removed from the soil, as well as the layer of partly decaying organic material (duff) up to a depth of approx. 10 cm into the humus layer; smaller plant roots had to be removed. The frames were placed onto the humus material and then covered approximately 2 cm deep with sieved (mesh size, 5 mm) humus material as freshly taken from a closely located site. Finally, the duff material and the litter were placed back onto the soil. The frames were kept in the soil for over one year, covering spring, summer, winter and the following spring. After recovery, the films were fully dried at room temperature and examined by scanning electron microscopy.

Scanning electron microscopy. LCAP films from the forest soil incubation were cut into 5 x 5 mm pieces with a scalpel and adhered to silver tape, each mounted onto carbon pads on aluminum stubs (12 mm diameter). These samples were then coated with a 6.5 nm layer of platinum using a sputter coater (Quorum Q150 R ES, Quorum Technologies, Lewes, United Kingdom). The morphological analysis of the film was conducted using a scanning electron microscope (Zeiss Auriga Cross Beam, Zeiss, Oberkochen, Germany), operated at 5 kV with the secondary electron detector.

Microcosm degradation assay. Biodegradation of polymers in forest soil was assessed under laboratory conditions through determination of CO_2-production as previously described [3, 4]. Briefly, 1g of forest soil was weighed into 25-mL culture vials and spiked with 50 mg of cellulose (control) or PHBV, PCL, HDPE, PE-2,18, PE-12,12, or PE-18,18. The polymer-spiked soil samples were prepared in triplicate and mixed thoroughly before the addition of sterile ddH_2O to achieve a moisture content equivalent to 70% of the soil's water holding capacity (WHC). As a reference for the basal respiration in the forest soil, non-polymer spiked soil samples were prepared, likewise in triplicate. The vials were closed with modified butyl-stoppers equipped with CO_2-traps filled with, initially, 0.1 M NaOH. The concentration of the NaOH was increased to 0.5 M after saturation was observed at early incubation time points (see main text, Results). The sample vials were incubated at 30°C and the NaOH sampled regularly. The CO_2 captured in the NaOH solution was quantified in a TOC-L (Shimadzu, Kyoto, Japan) and the results used to generate cumulative degradation curves. To counteract suspected nutrient limitation during degradation, 200 µL of a nutrient solution were supplied after 66 days. The nutrient solution provided 2 mM potassium phosphate, 0.125 mM magnesium sulfate, 10 mM ammonium chloride and µM-concentrations of various trace metals (see Table S1), as calculated based on the volume of total water content of the soil in the microcosms. Through nutrient addition, the moisture content was increased to 80% of the soil WHC.

DNA extraction and PCR. DNA was extracted from soil sub-samples after 164 days of incubation using a cetyltrimethylammonium bromide (CTAB)-based method[5]. To avoid PCR inhibition by soil constituents such as humic acids, the DNA was additionally cleaned using polyvinylpolypyrrolidone spin-columns before down-stream applications[6]. The 16S rRNA gene was amplified using primers Bakt341F (CCTACGGGNGGCWGCAG) and Bakt805R (GACTACHVGGGTATCTAATCC), targeting the V3 and V4 region of the gene [7]. The internal spacer region 2 (ITS2) was amplified using primers fITS7 (GTGARTCATCGAATCTTTG) and ITS4 (TCCTCCGCTTATTGATATGC) [8]. All primers were equipped with the appropriate adapters for Illumina sequencing platforms.

Amplicon sequencing analysis. The 16S rRNA gene amplicons were further processed as per the Illumina "16S Metagenomic Sequencing Library Preparation" protocol (Illumina, San Diego, CA, United States). Library preparation and sequencing on the MiSeq System (Illumina) were performed by Eurofins genomics (Konstanz, Germany). The sequencing reads were processed in R (v.4.3.2) utilizing packages dada2 (v.1.26.0) as described in a previously reported analysis pipeline [9]. Taxonomy was assigned to ASVs using the Silva database version 138.1 for bacterial 16S rRNA gene amplicons and the UNITE ITS database version 9 for fungal ITS amplicons [10, 11]. Microbial community analysis was performed in

R using packages pyhloseq (v.1.42.0) [12], Biostrings (v.2.66.0) [13], ape (v.5.7.1) [14], and vegan (v.1.8.8) [15]. Log-fold change analysis (lfc) was performed using R package ANCOMBC2 (v.2.0.2) with default settings and “struc_zero” set to FALSE [16]. For lfc analysis, datasets from all samples treated with the LCAP materials were grouped together, while datasets of cellulose-, PCL-, and PHBV-treated samples were considered as independent groups. Datasets of samples without plastic addition were used as a reference group together with datasets of samples provided with HDPE, due to their similar community composition and lack of substrate-induced CO_2 evolution in those samples. Prior to analysis, ASVs with less than 100 counts across 10% of samples were excluded to avoid over-estimation of rare taxa in the analysis. R packages ggplot(v.3.4.2) and ggsci(v.3.0.0) were utilized in data visualization [17].

Metagenome analysis. For metagenomic shotgun sequencing, the triplicate DNA samples of the respective treatments were combined and submitted as one sample per treatment for sequencing on an Illumina HiSeq device at Eurofins Genomics (Konstanz, Germany). Quality filtering of the resulting reads, metagenome assembly and analysis were conducted within the anvio (v.7.1) environment [18, 19]. Metagenome assembly was conducted using megahit (v. 1.2.9) with a minimum contig length of 1000bp. Mapping of the reads to the assembled contigs was done using bowtie2 (v.2.3.5) [20]. The anvio metagenome pipeline was further employed to create a contigs database, which includes the identification of open reading frames (ORFs) using Prodigal [21]. Amino acid sequences for identified ORFs were exported and analyzed further. The program “anvi-run-scg-taxonomy” was used to assign taxonomy to contigs based on single-copy core genes present in the metagenome with reference to the genome taxonomy database (GTDB) [22]. The program “anvi-profile” was used to obtain coverage information. The program ”anvi-estimate-scg-taxonomy” was used to estimate the taxonomic affiliation of bins based on single-copy core genes present in the assigned contigs.

Identification of putative plastic degrading enzymes in the metagenomes. Putative plastic degrading enzymes were identified by employing hidden markov models (HMMs) of previously reported plastic degrading enzymes, similar to a previously reported approach. [23]. Amino acid sequences of all plastic degrading enzymes reported in the PlasticDB were downloaded and grouped by the plastics they were reported to degrade [24]. Sequences of enzymes reported to degrade plastics polyethylene and polystyrene were combined. The grouped sequences were subsequently aligned using MAFFT (v.7.520) [25], followed by the creation of HMM motifs using HMMer (v.3.1b2) [26]. Results were pre-filtered to include hits with e-values <10E-3. A local blastP (v. 2.12.0) search was conducted using the PlasticDB sequences as reference database to identify the closest database match [27]. To obtain

information about possible secretion motifs, pre-filtered sequences were analyzed using SignalP-6 [28]. Finally, replicate hits that matched multiple HMM motifs were removed, resulting in a dataset of unique metagenomic protein sequences with significant HMM-based similarity to previously reported plastic degrading enzymes. To obtain a more concise list of candidates potentially capable of LCAP depolymerization, the dataset was further analyzed based on normalized contig coverage to account for differences in total metagenome size between samples, in order to identify candidate enzymes with increased coverage in LCAP-treated samples. To this end, the sequences of candidate enzymes were analyzed via principal component analysis (PCA), using R packages FactoMineR (v. 2.11) and factoextra (v. 1.0.7) [29], to identify the most differentially covered sequences in the dataset. Candidate sequences with a negative value on the PC2-axis and above a PC1-axis threshold of 5 were selected for manual curation based on the predicted presence of a secretion signal, a more stringent cut-off for the e-value of the HMMer search score (<10E-5), as well as their BlastP e-value (<10E-10).

Structural analysis and docking simulations. The structural model for LCPH1 used for docking simulations was obtained using the Alphafold3 web server [30]. The active site of LCPH1 was predicted based on the obtained structural model using the ProFunc web server [31]. Ligand structures used for modelling were downloaded from PubChem in the case of the β-lactam antibiotics Ampicillin and Penicillin. Structures of polyester oligomers (PE-12,12 dimer & PCL trimer) were obtained from their respective SMILES specification using Open Babel (v. 3.1.1) [32, 33]. The protein and ligand structures were prepared for docking using Autodock Tools (wihtin MGLTools v. 1.5.7) [34]. The grid-box was placed centred on the active site serine and its size adjusted to fit the respective ligand. The maximum amount of active torsions was allowed for each ligand and docking was performed using the genetic Lamarckian algorithm. The search was conducted using a population size of 150, $5x10^7$ energy evaluations, and $2.7x10^4$ generations in 100 individual runs.

In case of the oligomeric ligands, the docking simulation did not converge into clear clusters. Therefore, the best-scoring docking solution with close proximity of the central ester bond to the catalytic residues was chosen for refinement by repeated docking with reduced active torsions. For the PE-12,12 dimer, the number of active torsions was reduced from 30 to 16 and for the PCL trimer from 21 to 12, thereby reducing the complexity of the search. After refinement, clear clusters were obtained for the oligomeric ligands. The β-lactam ligands formed clear clusters without refinement being required.

For each docking simulation, distances between the nucleophilic serine and the ligand carbonyl carbon, as well as the attack angle (Bürgi-Dunitz angle) of the nucleophilic serine

were determined using ChimeraX (v. 1.8) [35]. The catalytically most favourable docking solution was exported for visualization and further analysis.

Multiple sequence alignment. The LCPH1 amino acid sequence was aligned to homologous proteins from the PlasticDB database (IDs 91 and 158) as well as type C beta lactamases p99 of *Enterobacter cloacae* (PDB entry 1XX2) and BlaEC of *Escherichia coli* (PDB entry 9C6P), family VIII type esterases EstB of *Burkholderia gladioli* (PDB entry 1CI8), and metagenomic enzymes EH7 (PDB entry 7PP3) and Est-Y29 (PDB entry 4P6B). Multiple sequence alignment was conducted using the MAFFT web server using default settings except for the addition of further homologous sequences through the implementation of DASH [36]. The sequences added by DASH for improved alignment were omitted in the final result. The alignment was visualized using Biopython and Matplotlib [37, 38].

Heterologous expression of putative plastic degrading enzymes. The LCPH1-coding gene without its secretion signal, with C-terminal His-tag, and codon-optimized for heterologous expression in *E. coli*, was ordered in a pET-26b(+) expression vector at BioCat (Heidelberg, Germany). The vector was transformed into *E. coli* expression strain Rosetta Gami 2 DE3, and the insert confirmed via restriction digest. For expression of the target enzyme, 50 mL over-night cultures were prepared using LB-medium supplemented with 50 mg/L Kanamycin (Kn_{50}) and 20 mg/L Chloramphenicol (Cm_{20}). Kn-resistance was conveyed by the pET-26b(+) vector, while Cm-resistance was conveyed by a Rosetta Gami 2 plasmid encoding rare t-RNAs. Overnight cultures were inoculated with transformed cells and grown at 37 °C and 200 rpm horizontal shaking in 200 mL Erlenmeyer flasks. The cultures were then used to inoculate 1 L cultures containing 950 mL of LB medium with additional 10 mM Glucose and Kn_{50} and Cm_{20}. Glucose was added to prevent basal expression prior to induction by carbon catabolite repression [39]. The cultures were then grown to an OD600 of 0.6-1.0 before induction with 0.4 mM IPTG. The cultures were then transferred to a cooled incubator for expression at 18 °C and 100 rpm for 18-20 h.

Cells were centrifuged at 5,000 x *g* for 15 min and washed twice with 50 mM PBS (pH 7.4, 300 mM NaCl). Cell pellets were frozen in liquid nitrogen and stored at -80 °C until further processing. For cell lysis the cells were resuspended in 50 mM PBS (pH 7.4, 300 mM NaCl) supplemented with 10 mM imidazole and 0.1% Triton-X. Triton-X was used to aid in protein solubility during the lysis and extraction process. Additionally, cOmplete™ ULTRA proteinase inhibitor tablets (Roche, Basel, Switzerland) and DNAse I were added to the lysis mixture. Lysis was achieved via ultra-sonification using a Sonoplus HD 2070.2 with a VS70T probe (Bandelin, Berlin, Germany) at an amplitude of 25% with 1s active and 3 s inactive intervals for 8 min, while the sample was cooled on ice. Cell debris and unbroken cells were collected by centrifugation at 16,000 x *g* for at least 30 min. The supernatant was then filtered through

a 0.45 µm pore-size cellulose acetate syringe filter and the cell-free extract was purified via immobilized metal ion chromatography (IMAC) using a 5 mL HisTrap Excel column (Cytiva, Marlborough, MA, United States) attached to a Äkta-Go FPLC device (Cytiva, Marlborough, MA, United States). Elution of column-bound protein was achieved by Imidazole gradient fractionation over 6 column volumes. Collected fractions were analyzed via SDS-PAGE to confirm presence of the target protein. Purified samples were desalted using 15-kDa Amicon centrifugal filters (Merck Millipore, Burlington, MA, United States). Protein concentration after desalting was determined using the Qubit4 Protein Broad range kit (Thermo Fisher, Waltham, MA, United States).

Enzyme activity testing. All enzyme assays were carried out at pH 7.4 in 50 mM PBS containing 300 mM NaCl and 0.1% Triton-X. Triton-X, apart from aiding in protein solubility, was used in plastic degradation assays for its surface active properties, ensuring a homogenous dispersion of milled plastic particles in the reaction mixture. To generally assess esterase activity, the purified protein was incubated in cuvettes at a concentration of 1 µM with 0.5 mM of *p*-nitrophenyl butyrate in a total volume of 1 mL; upon ester-bond cleavage, *p*-nitrophenol is released, which was followed at 397 nm [40].

Polyester depolymerase activity was assessed discontinuously by following the formation of monomers. For determination of PCL-depolymerase activity, 2.5 µM of enzyme was incubated with 5 ± 0.1 mg PCL powder (≤ 100 µm diameter particle size) in 1 mL assay volume in 1.5 mL microcentrifuge tubes. The reactions were incubated at 30°C with horizontal shaking at 120 rpm for up to 24 h. Sub-samples of 200 µL were taken at multiple timepoints, and the reaction in the sub-samples stopped by addition of 10% (v/v) of 1 M H_2SO_4. Acidified samples were centrifuged at maximum speed in a table-top centrifuge and the particle-free supernatant transferred to a HPLC vial.

For determination of LCAP-depolymerase activity, assays were also carried out in 1.5 mL microcentrifuge tubes, in either 0.5 mL or 1 mL total assay volume per reaction. Tubes contained 3 ± 0.1 mg PE-2,18 powder (≤ 100 µm diameter particle size) in 0.5 mL assay volume and purified enzyme was added to a concentration of 2.5 µM. In assays conducted with 1 mL volume, 5 ± 0.1 mg of PE-2,18 powder was used. Samples were incubated at 30°C under horizontal shaking at 120 rpm for up to 72 h. For samples with 1 mL volume, sub-samples of 200 µL were taken at multiple timepoints, and the reactions in the sub-samples were stopped by either addition of an equal volume of isopropanol (200 µl) for samples analyzed by HPLC-MS, or by addition of 1 M H_2SO_4 to a concentration of 10% (v/v) for samples analyzed by HPLC-RID. For samples with 0.5 mL volume, the entire sample was used. Assay reactions were conducted in triplicate or in single samples, as specified. Assay buffer with plastic but without enzyme and heat-inactivated enzyme samples supplemented

with plastic were included as negative controls. The activity LCPH1 for LCAP degradation was compared directly to that of *Ideonella sakaiensis* PETase (IsPETase) [41], which was expressed and purified using a pET-21b expression vector, kindly provided by the research group of Prof. Dr. Uwe Bornscheuer at Universität Greifswald.

Formation of monomer caprolactone was determined with a Shimadzu LC-20 HPLC system (Shimadzu, Kyoto, Japan) equipped with refractive index detector (RID), using 1% (v/v) H_2SO_4 in distilled water as mobile phase with a flowrate of 0.6 mL/min and a Rezex™ RHM-Monosaccharide H+ (8%) Ion Exclusion HPLC Column (Phenomenex, Torrance, CA, United States) for separation.

LCAP monomer formation was quantified by detection of both PE-2,18-monomers, ethylene glycol and 1,18-octadecanedioate. Ethylene glycol concentrations were determined via HPLC-RID as described for caprolactone. Octadecanedioate concentrations were determined via HPLC-ESI-MS as previously described [42]. Briefly, an equal volume of MS-grade isopropanol was added to each sub-sample taken form an enzyme reaction, and the samples were mixed and incubated at 37 °C for at least 30 min in order to facilitate dissolution of octadecanedioate and precipitation of protein. Samples were then centrifuged in a table-top centrifuge at maximum speed and the particle-free supernatants were transferred to HPLC vials. Separation and detection of 1,18-octadecanedioate was achieved using MilliQ-water acidified with 0.1% (vol/vol) formic acid and acetonitrile as mobile phases, a Hypersil ODS C18 HPLC column (Thermo Fisher, Waltham, MA, United States), and ESI ionization in the negative mode. All monomers were quantified against authentic standards.

Degradation of β-lactam antibiotics Penicillin G and Ampicillin by LCPH1 was followed in 0.5 mL reactions with 500 µM of the respective antibiotic and 2.5 µM purified LCPH1. The reactions were incubated in glass HPLC vials at room temperature and without shaking in the autosampler of a Shimadzu LC-20 HPLC system (Shimadzu, Kyoto, Japan) equipped with a diode array detector (DAD). Samples were injected at 1 – 2 h intervals for 24 h. Penicillin G and Ampicillin concentrations were determined against authentic standards using a gradient of MilliQ-water with 10 mM ammonium formate (eluent A) and 10% MeOH and (eluent B) 100% MeOH, starting at 45% A at a flow-rate of 0.5 mL/min, and a Nucleosil™ 100-5 C18 column (Machery-Nagel, Düren, Germany); the compounds were detected at 254 nm.

For detection of the intramolecular hydrolysis products from β-lactamase activity of LCPH1 on Ampicillin or Penicillin G, reactions contained 5 µM LCPH1 (or PETase as control) in PBS and 500 µM of the antibiotic. Sub-samples were taken from reactions and analysed when employing a Shimadzu LC-40 HPLC system coupled to a MS2020 ESI-MS. Separation was

achieved using a Hypersil ODS C18 HPLC column (Thermo Fisher) and gradient elution, with (eluent A) MilliQ-water containing 10 mM ammonium formate and 10% (v/v) acetonitrile, and (eluent B) acetonitrile containing 10 mM ammonium formate and 10% (v/v) water; the gradient started at 4 min from 0% to 75% eluent B at 9 min, at a flow rate of 0.3 ml/min. Target compounds were detected by their molecular ions in the positive ionization-mode ($[M+H]^+$): Ampicillin at *m/z* = 350 Da and ampicilloic acid at *m/z* = 368 Da, and Penicillin G at *m/z* = 335 Da and penicilloic acid at *m/z* = 353 Da.

B. Supplementary Figures

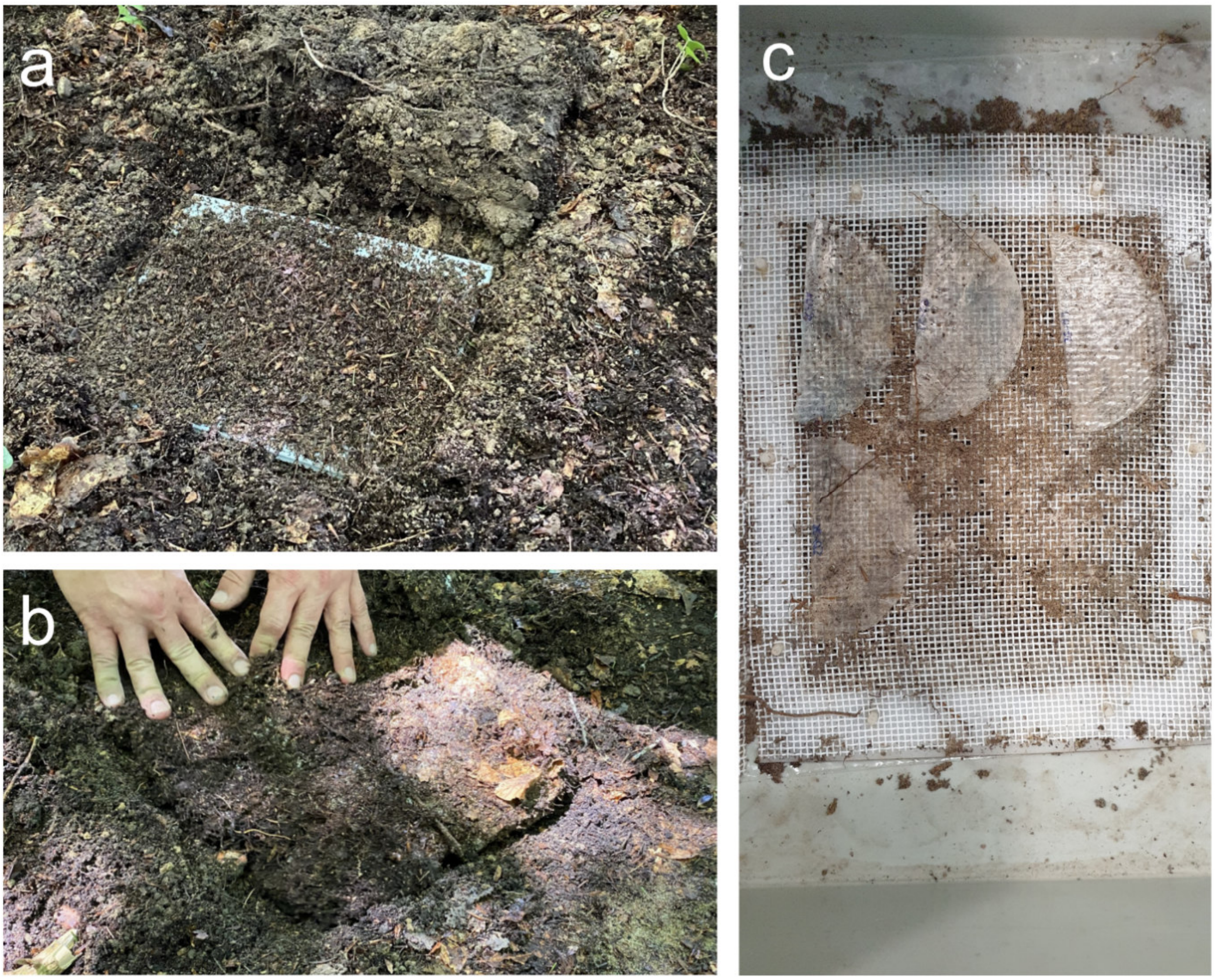

Figure S1. Photographs illustrating the placement of the LCAP films mounted into 'HYDRA test frames' in forest soil. For the test frames, see Lott et al. [2]. Shown are images of the test frames placed under a layer of sieved humus (**a**) and forest top-soil (**b**), and of the macroscopical appearance of the LCAP films after recovery and opening of the test frames (**c**). For scanning electron microscopy (SEM) images of the surfaces of the plastic films before and after incubation, see Fig. S2, and in the main text, Fig. 1.

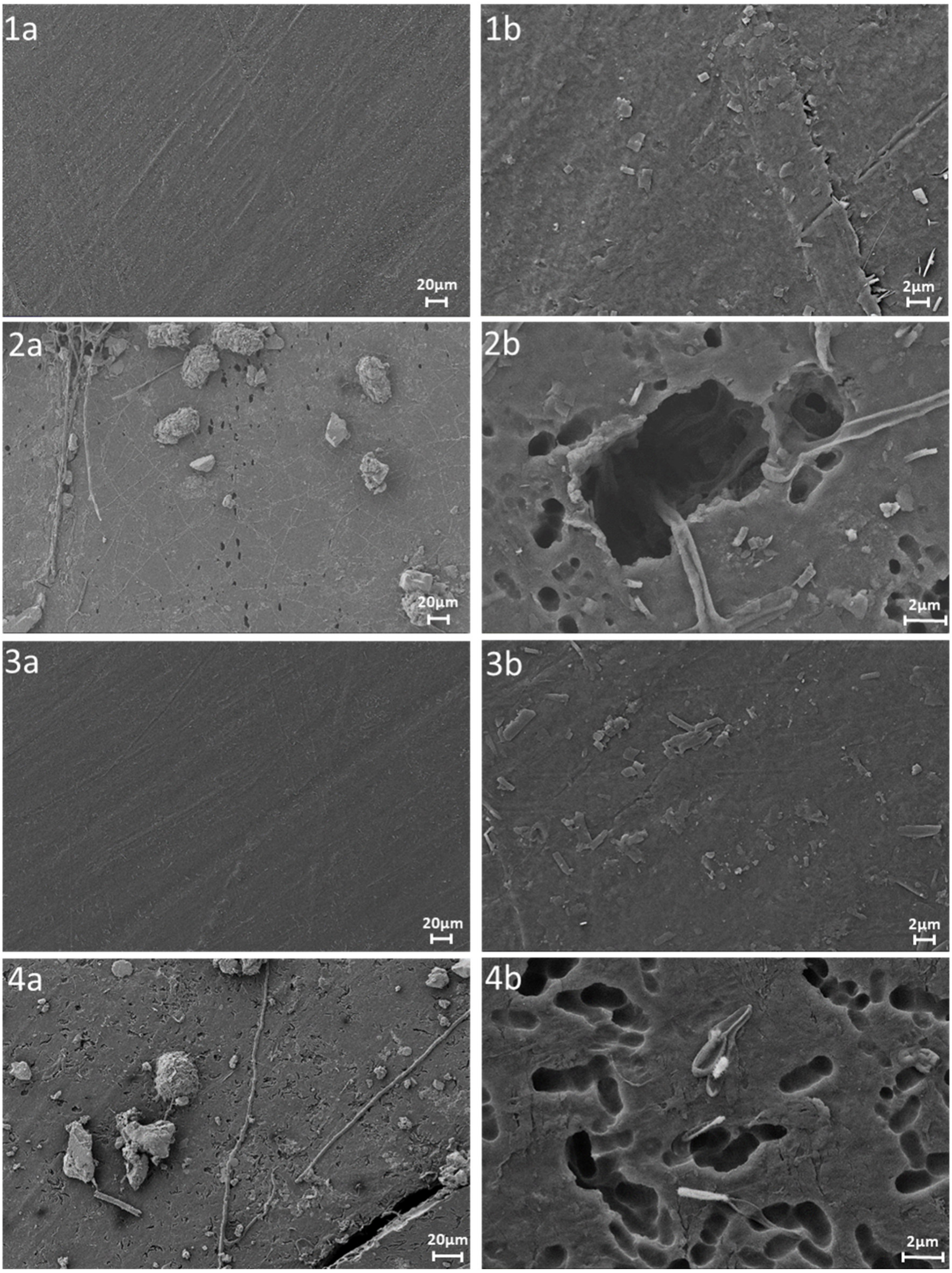

Figure S2. Scanning electron microscopy (SEM) images of PE-12,12 and PE-18,18 films incubated in pristine forest soil for about one year. The images were taken at the same position with two different magnifications, as indicated by 20-µm and 2-µm scale bars. The structural damage on the PE-12,12 and PE-18,18 films after incubation (images **2a** and **2b**, and **4a** and **4c**, respectively), which was absent for non-incubated foils (images **1a** and **1b**, and **3a** and **3b**, respectively), most likely resulted from microbial degradation of the plastic material. See also the additional images in the main text (Fig. 1).

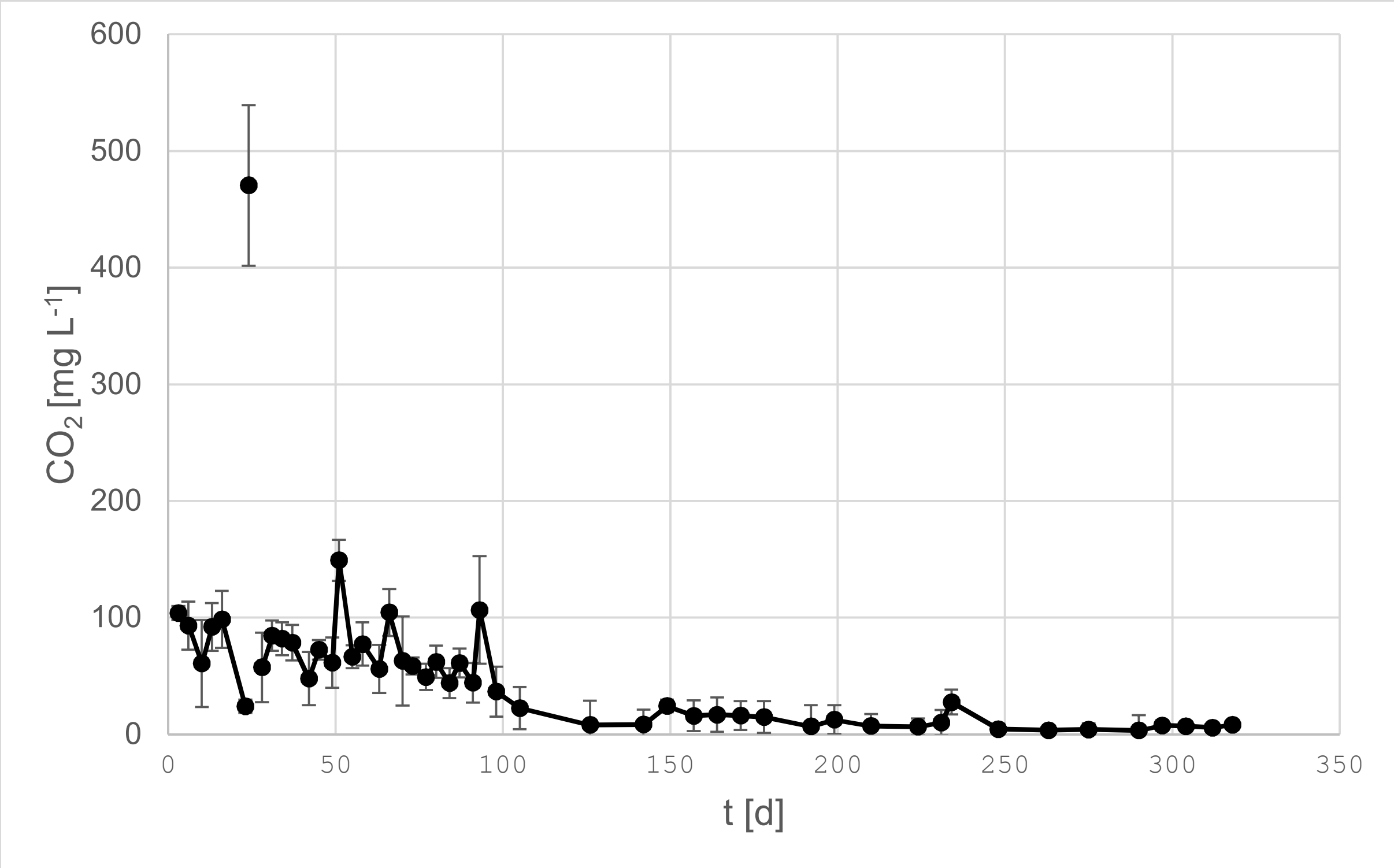

Figure S3. Average daily CO_2 rates in soil blank microcosms. Rates were determined by division of the measured CO_2 concentration at a given time point by the amount of days since the previous measurement. Error bars show the standard deviation of the triplicate microcosms. The outlier observed at 24 d relates to the observed saturation of the NaOH solution at the previous time point, likely resulting in accumulation of CO_2 in the microcosm headspaces.

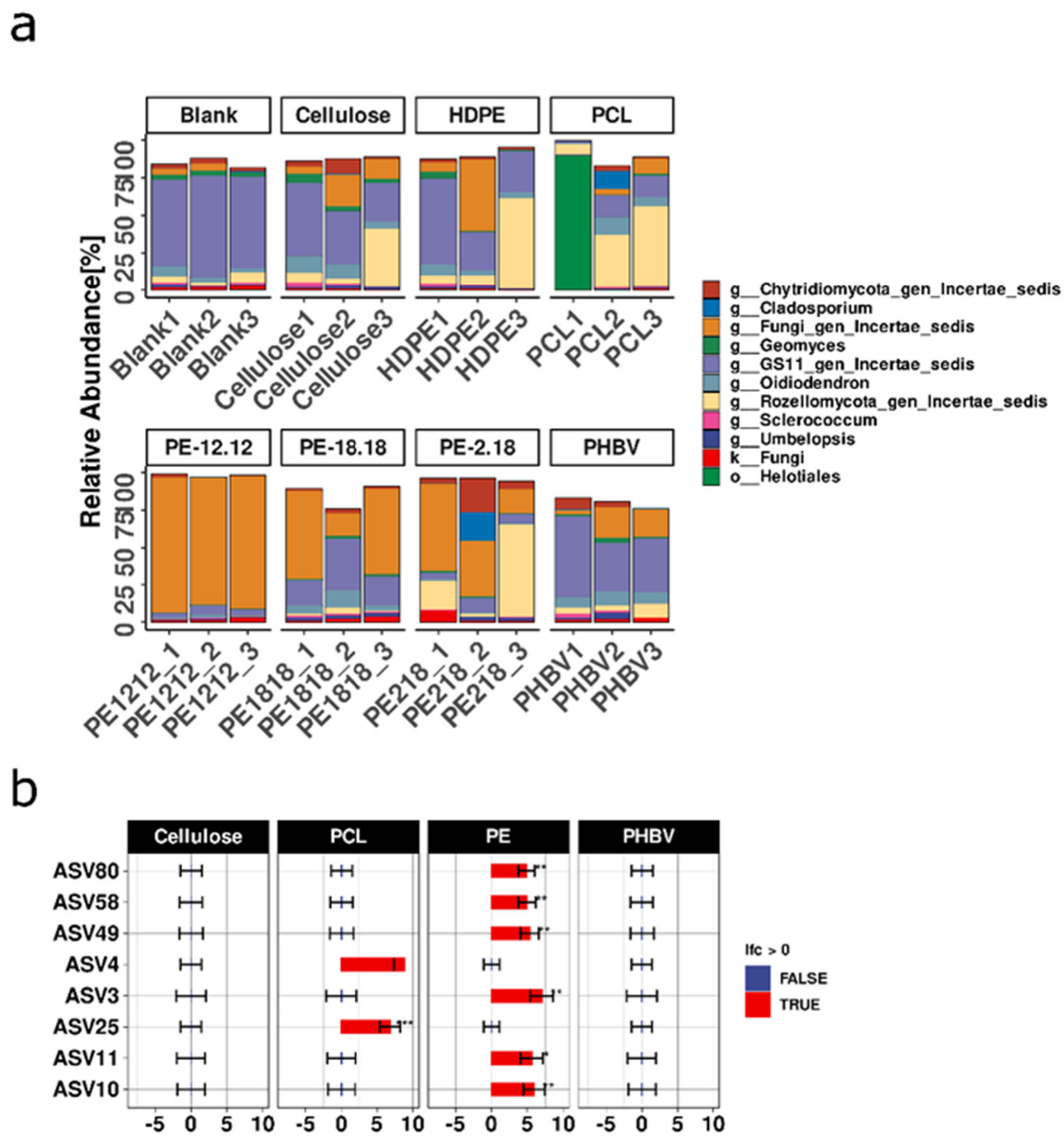

Figure S4. Bar plots illustrating the ITS gene diversity (genus level) of the fungal communities in soil samples incubated with the different polymers and Log-fold change (LFC) analysis for differentially abundant taxa. (**a**) The community composition is shown for all replicate treatments. ASVs assigned to the same genus were combined. When a genus could not be determined with sufficient confidence, the last taxonomic level assigned is indicated. Only ASV groups with a mean relative abundance of >1% are depicted. (**b**) Non-spiked microcosms (blanks) and the HDPE-spiked microcosms were grouped together as reference. Likewise, all microcosms treated with the three types of LCAPs were grouped together, while replicates of the cellulose-, PCL- and PHBV-treatments formed separate groups. To reduce the impact of sequencing artifacts and extremely rare ASVs, only ASVs with a count of ≥100 across 10% of samples were included in the analysis. Red bars indicate a significant increase in a taxon's abundance compared to the control condition. Blue bars indicate a reduction. Statistical significance of the LFC is indicated next to the bars (*: P ≤ 0.05, **: P ≤ 0.01, ***: P ≤ 0.001, ****: P ≤ 0.0001).

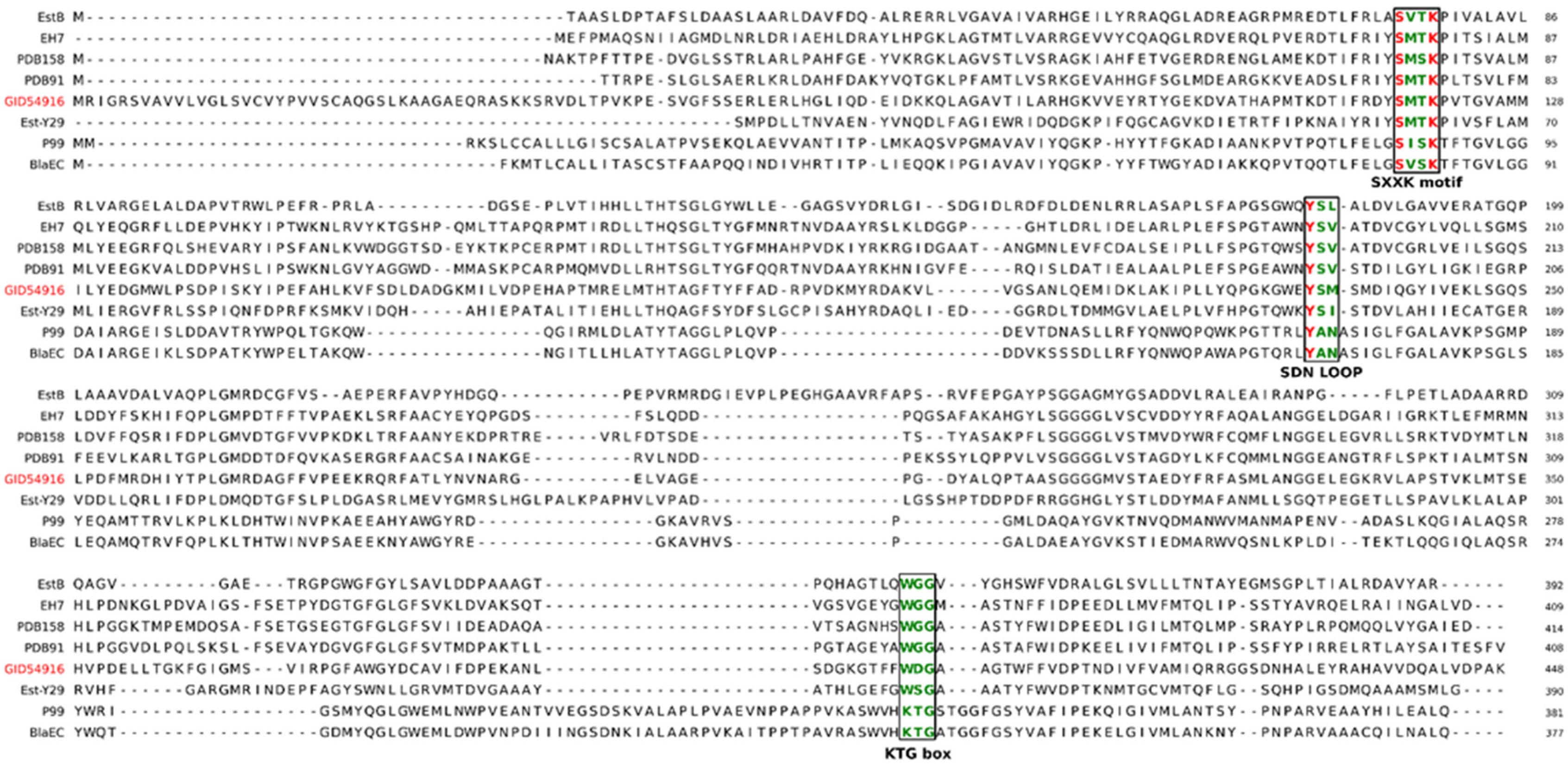

Figure S5. Multiple sequence alignment of LCPH1 and other family-VIII esterases. Sequences used for comparison were EstB from *Burkholderia gladioli*, Est-14 (GeneBank KY483644), the PlasticDB (PDB) entries 91 and 158 and class C β-lactamases p99 from *Enterobacter cloacae*, BlaEC from *Escherichia coli*, and the metagenomically sourced penicillin-binding β-lactamase homolog Est-Y29. The key conserved regions are highlighted in green, while residues making up the catalytic triad are highlighted in red.

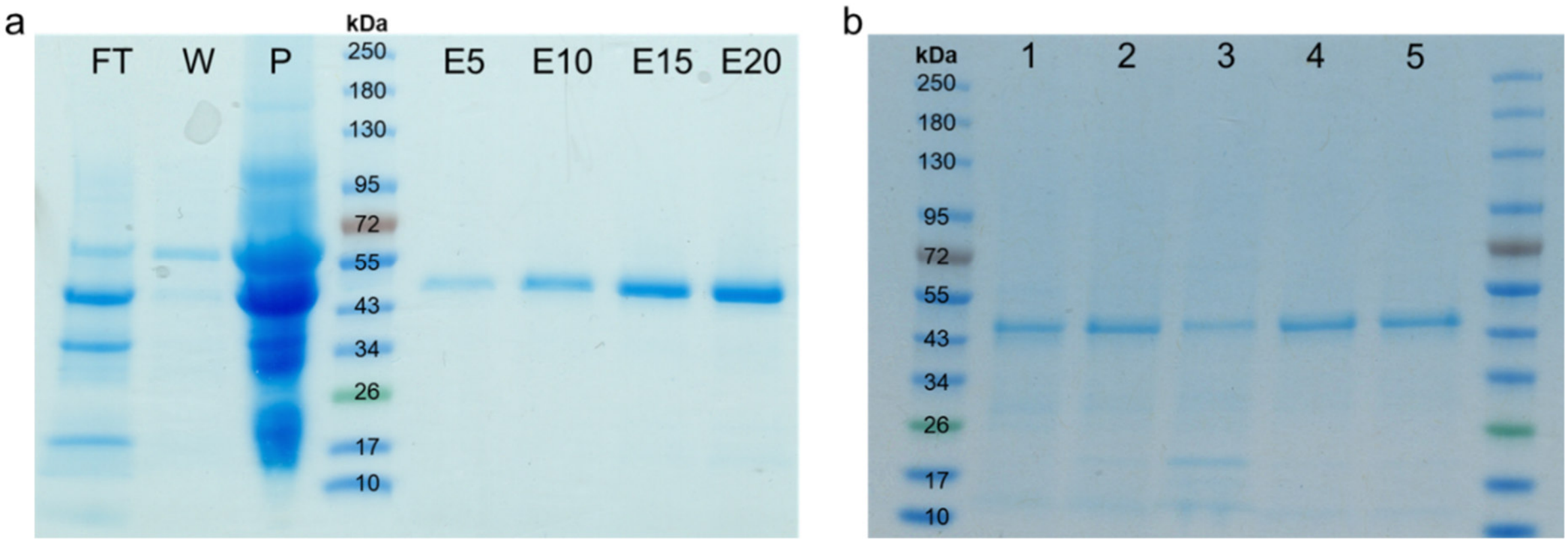

Figure S6. SDS-PAGE analysis of the heterologous expression and purification of His-tagged LCPH1. (**a**) Representative gel showing the different fractions collected during the process. Loaded were the His-Trap column flow-through (FT), the collected wash fraction (W), the proteins present in the cell pellet (P), and the protein eluted from the His-Trap column (E) when different sample volumes were loaded (5, 10, 15 and 20 µL). (**b**) SDS-PAGE of all GID54916 preparations used in this study (preparations 1-5, adjusted each to 2.5 µM GID54916). The predicted size of GID54916, including His-Tag, is 46.3 kDa.

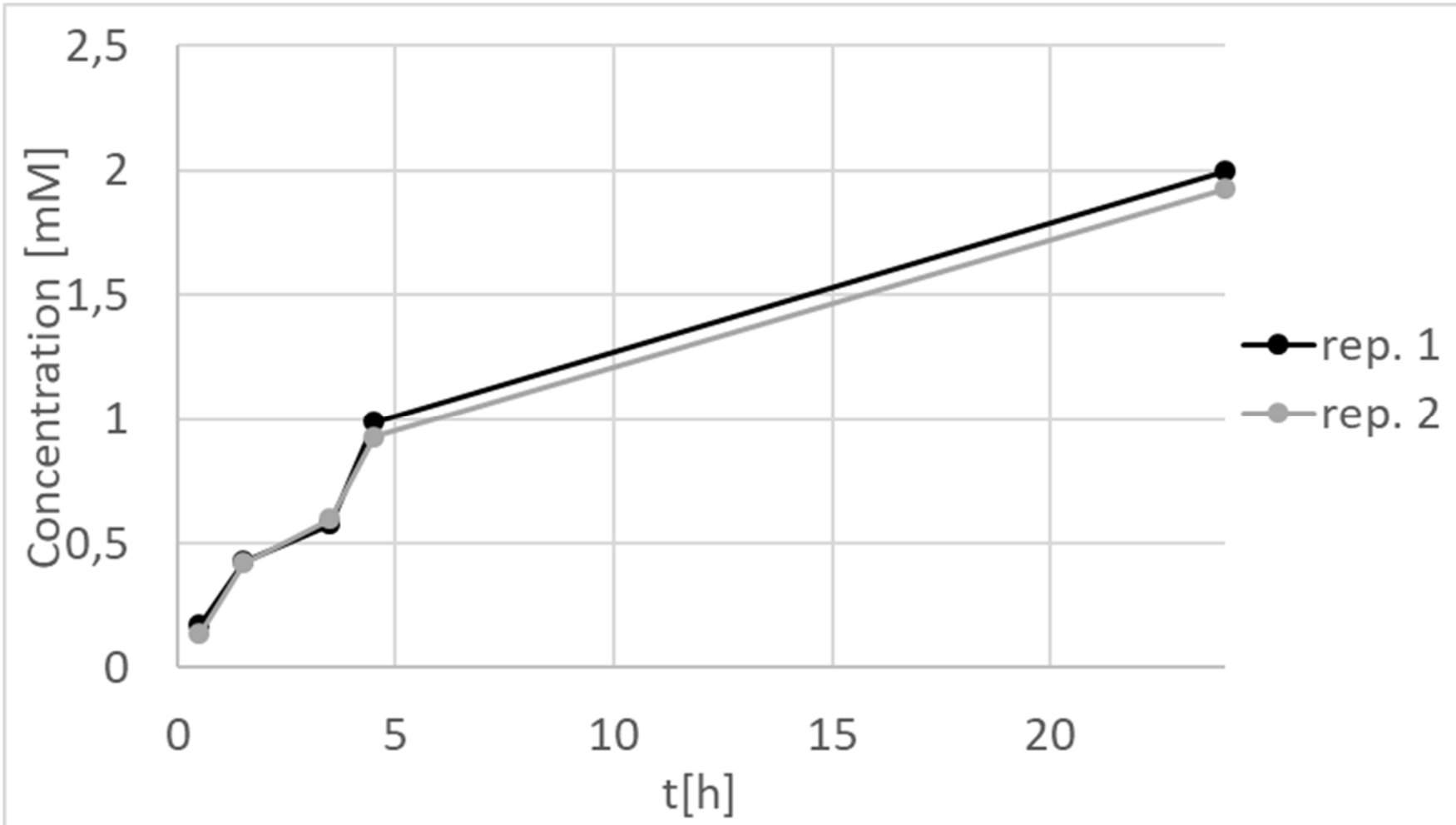

Figure S7. Discontinuous enzyme assay demonstrating ε-caprolactone monomer release from PCL plastic powder by recombinantly produced LCPH1. Reactions contained 5 mg of PCL powder and 2.5 µM enzyme in 1 mL PBS buffer including 0.1% (v/v) Triton X-100. Two replicate reactions were set up and samples of 200 µL taken at 5 time points over 24 h total incubation time; reactions in the samples were stopped by addition of 10% (v/v) of 1 M H_2SO_4. The formation of ε-caprolactone was quantified by HPLC-RID against authentic standard.

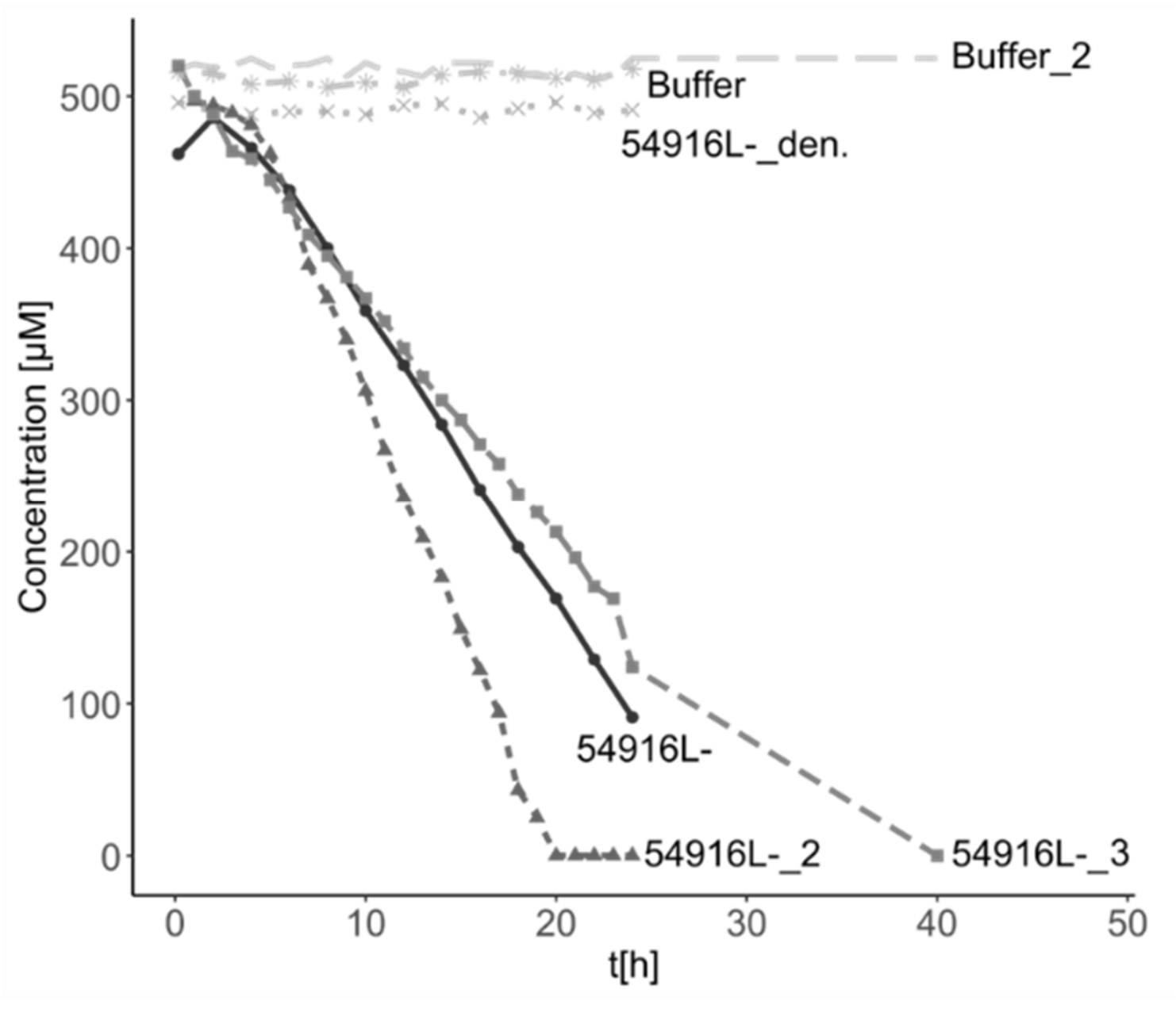

Figure S8. Discontinuous enzyme assay demonstrating the disappearance of Penicillin G by LCPH1 (GID54916). Reactions contained 500 µM Penicillin G and 2.5 µM LCPH1 from three individual enzyme preparations (expressions 1-3), each in PBS in 0.5 mL total volume. Heat-denatured LCPH1 and PBS buffer without added enzyme (buffer) served as controls. Sub-samples were taken at the intervals indicated and analysed for substrate disappearance by HPLC-DAD.

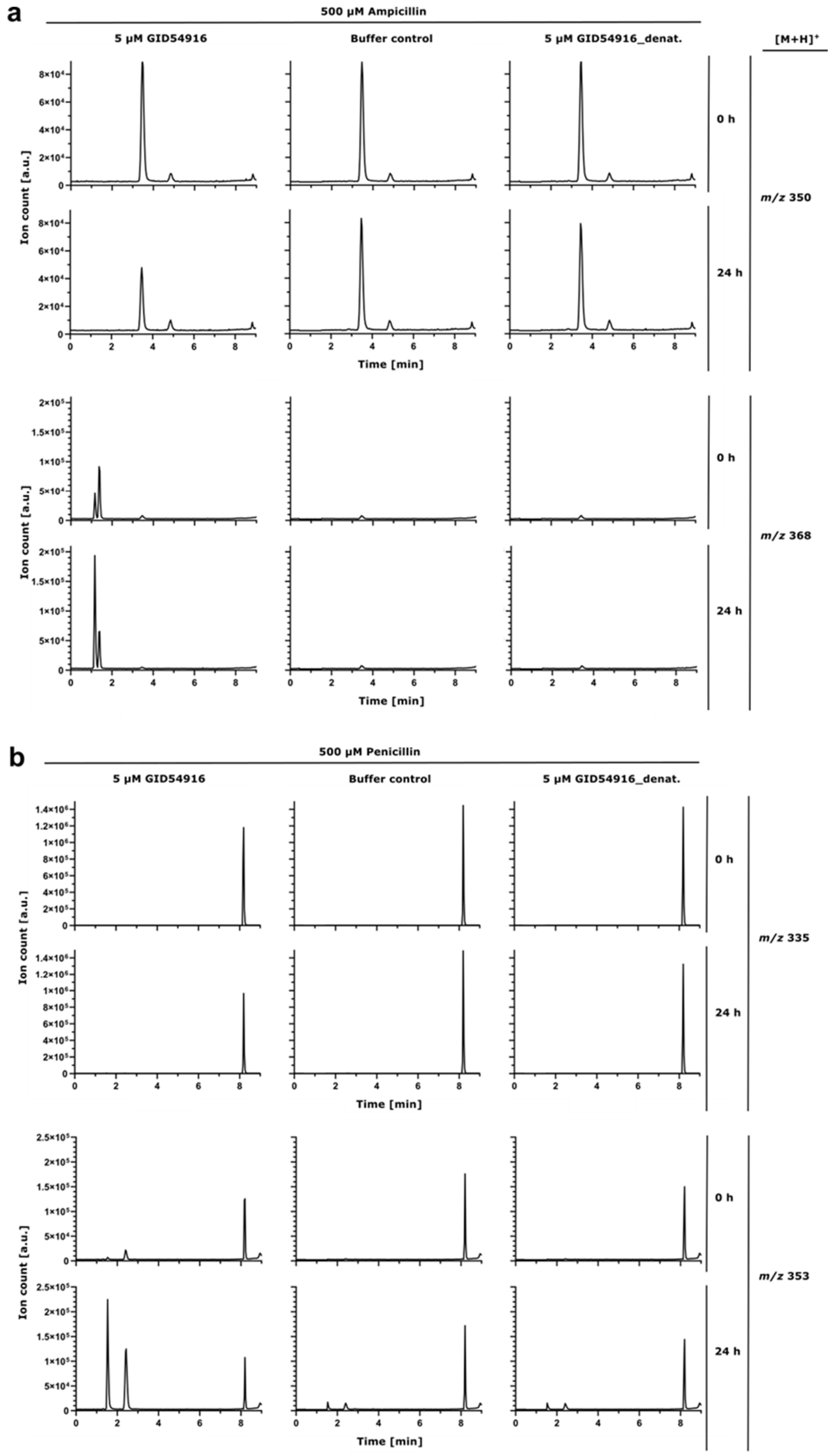

Figure S9. Confirmation by HPLC-ESI-MS of hydrolysis of Ampicillin and Penicillin G by LCPH1. Reactions contained 5 µM LCPH1 in PBS and 500 µM Ampicillin (**a**) or Penicillin G (**b**). Target compounds were detected by their molecular ions in the positive ionization-mode: Ampicillin at *m/z* = 350 Da with a peak at retention time 3.5 min, and Penicillin G at *m/z* = 335 Da with a peak at 8.2 min. The intramolecular hydrolysis products from opening of the β-lactam ring, ampicilloic acid at *m/z* = 368 Da and penicilloic acid at *m/z* = 353 Da, respectively, were detected as two peaks each, representing the two C5 epimers formed (*5R*,*6R*- and *5S*,*6R*-epimers), for ampicilloic acid at 1.2 min and 1.4 min, and penicilloic acid at 1.5 and 2.4 min retention time. Substrates in PBS without enzyme (buffer control) or with heat-denatured LCPH1, served as negative controls. The reactions were analyzed at the start and after 24 h of incubation time.

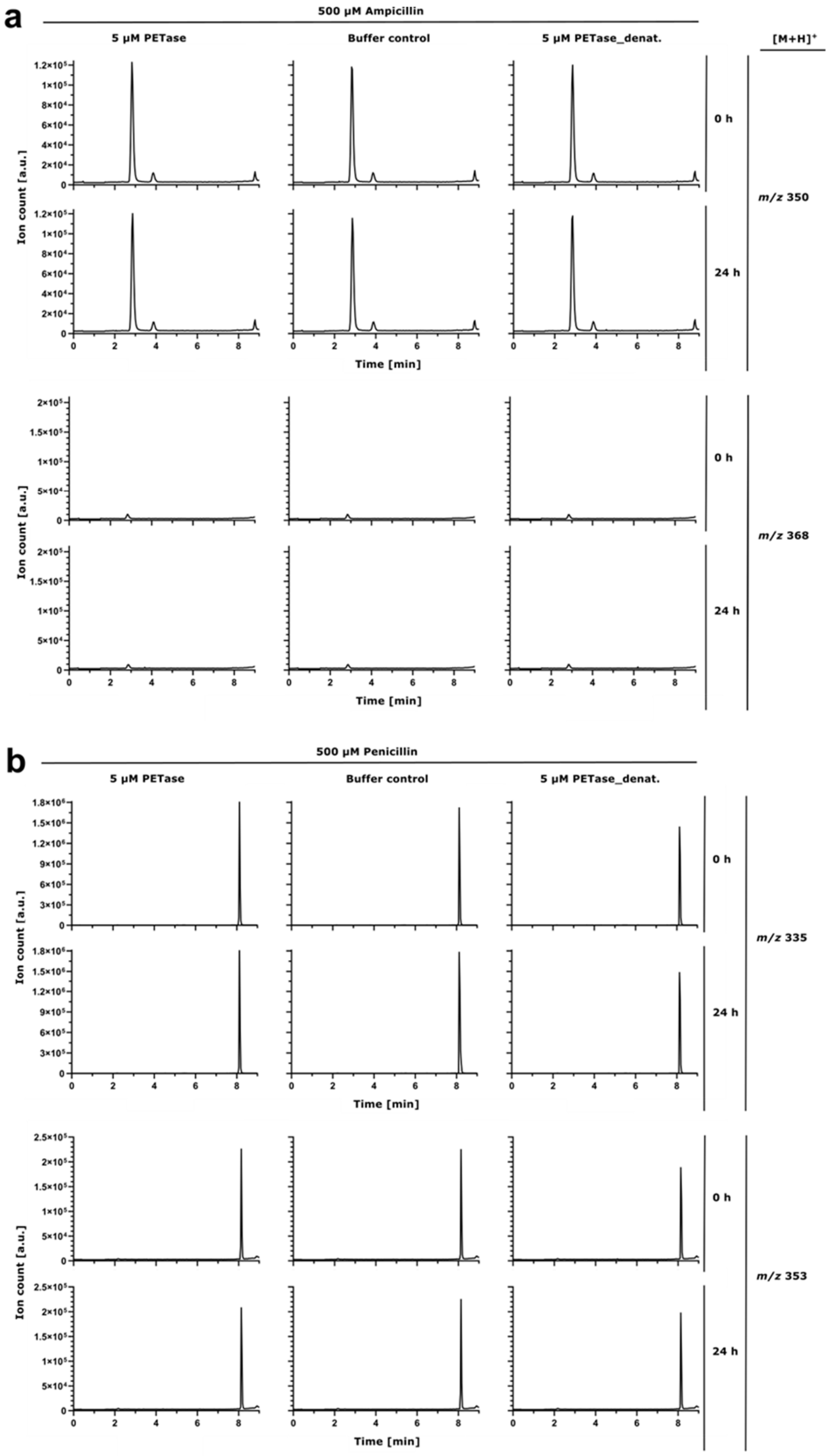

Figure S10. Confirmation by HPLC-ESI-MS that PETase cannot hydrolyze Ampicillin or Penicillin G. Reactions contained 5 µM of recombinantly produced PETase in PBS and 500 µM Ampicillin (**a**) or Penicillin G (**b**). The target compounds were detected as specified in the legend of Figure S9. The substrates in PBS without enzyme (buffer control) or with heat-denatured PETase, served as controls. The reactions were analyzed at the start and after 24 h of incubation time.

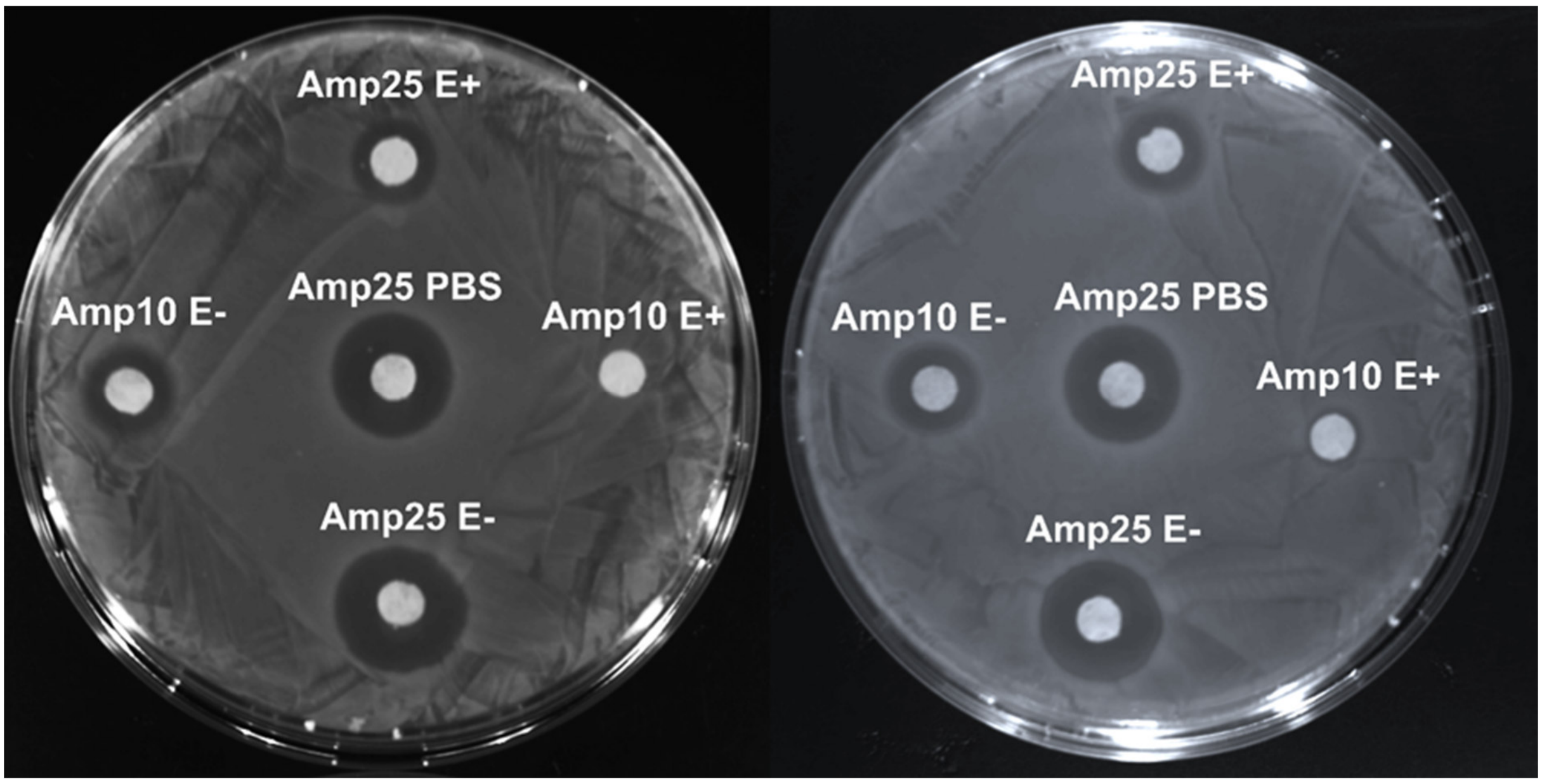

Figure S11. Ampicillin disc diffusion assay with and without LCPH1 **exposure.** Filter paper discs supplemented with 10 or 25 µg of Ampicillin were incubated over night at 30°C with 20 µL of 5 µM LCPH1 in PBS buffer (E+), 5 µM heat-denaturation LCPH1 (E-), or PBS buffer without enzyme (PBS). The discs were placed on Mueller-Hinton agar plates on which Ampicillin-sensitive *E. coli* K12 cells had been plated. Plates were incubated over night at 37°C and then photographed. Inhibition zones were diminished when discs were exposed to active LCPH1 for both Ampicillin concentrations tested.

C. Supplementary Tables

Table S1. Analysis of the utilized forest soil according to Albrecht Standard. The analyses were conducted by Geobüro Christophel (Lauterhofen, Germany).

Parameter	*Value*	*Units*
Cations		
Potassium	179	kg/ha
Sodium	28	kg/ha
Calcium	1193	kg/ha
Magnesium	374	kg/ha
Anions		
Sulfur	3	ppm
Phosphorus (available)	3.1	kg/ha
Phosphorus (stored)	151	kg/ha
Trace elements		
Boron	0.6	ppm
Iron	794.4	ppm
Manganese	103.3	ppm
Copper	1.1	ppm
Zinc	16.8	ppm
General soil data		
pH (H_2O)	4.3	
Total CEC*	13.3	mmol/100g
Humus Content	15.4	%
Total Nitrogen	0.52	%
C/N Ratio	17.2	

*Cation exchange capacity

Table S2. Concentrations of trace metals added to the soil microcosms. The listed final concentrations refer to the total water content of the soil based on determined water content and additionally supplied water including the nutrient solution (for further information, see main text).

**Trace Minerals and Salts**	**Final concentration [µM]**
$Na_2EDTA \cdot 2H_2O$	13.40
$FeCl_2 \cdot 4H_2O$	7.20
$ZnCl_2$	0.35
$MnCl_2 \cdot 4H_2O$	0.15
H_3BO_3	4.85
$CoCl_2 \cdot 6H_2O$	0.84
$CuCl_2 \cdot 2H_2O$	0.06
$NiCl_2 \cdot 6H_2O$	0.08
$Na_2MoO_4 \cdot 2H_2O$	0.12
$CaCl_2 \cdot 2H_2O$	6.80

Table S3. Taxonomic affiliation of differentially abundant ASVs. The ASVs were indicated in the Log-fold change (LFC) analysis, as shown in Figure 4 of the main text. BCP, Burkholderia-Caballeronia-Paraburkholderia.

taxon	group	lfc	Se	signif	Phylum	Class	Order	Family	Genus
ASV10	Cellulose	2.956	0.771	*	Proteobacteria	Alphaproteobacteria	Micropepsales	Micropepsaceae	Family_Micropepsaceae
ASV10	LCAP	5.636	0.614	****	Proteobacteria	Alphaproteobacteria	Micropepsales	Micropepsaceae	Family_Micropepsaceae
ASV11	LCAP	3.768	0.821	**	Acidobacteriota	Acidobacteriae	Acidobacteriales	Acidobacteriaceae	Terracidiphilus
ASV112	Cellulose	2.728	0.682	*	Proteobacteria	Alphaproteobacteria	Micropepsales	Micropepsaceae	Family_Micropepsaceae
ASV112	LCAP	5.002	0.549	****	Proteobacteria	Alphaproteobacteria	Micropepsales	Micropepsaceae	Family_Micropepsaceae
ASV114	LCAP	4.599	0.825	****	Proteobacteria	Alphaproteobacteria	Micropepsales	Micropepsaceae	Family_Micropepsaceae
ASV118	LCAP	3.381	0.798	**	Acidobacteriota	Acidobacteriae	Subgroup 2	Order_Subgroup 2	Order_Subgroup 2
ASV121	Cellulose	3.064	0.737	**	Acidobacteriota	Acidobacteriae	Acidobacteriales	Acidobacteriaceae	Terracidiphilus
ASV121	LCAP	4.306	0.590	****	Acidobacteriota	Acidobacteriae	Acidobacteriales	Acidobacteriaceae	Terracidiphilus
ASV121	PHBV	3.284	0.744	**	Acidobacteriota	Acidobacteriae	Acidobacteriales	Acidobacteriaceae	Terracidiphilus
ASV127	PHBV	4.305	1.110	*	Proteobacteria	Gammaproteobacteria	Burkholderiales	Burkholderiaceae	BCP
ASV136	LCAP	4.532	0.787	****	Proteobacteria	Alphaproteobacteria	Micropepsales	Micropepsaceae	Family_Micropepsaceae
ASV137	Cellulose	4.683	0.351	****	Proteobacteria	Alphaproteobacteria	Sphingomonadales	Sphingomonadaceae	Sphingomonas
ASV137	LCAP	4.803	0.304	****	Proteobacteria	Alphaproteobacteria	Sphingomonadales	Sphingomonadaceae	Sphingomonas
ASV137	PHBV	4.258	0.357	****	Proteobacteria	Alphaproteobacteria	Sphingomonadales	Sphingomonadaceae	Sphingomonas
ASV142	Cellulose	4.900	0.678	****	Proteobacteria	Alphaproteobacteria	Caulobacterales	Caulobacteraceae	Caulobacter
ASV142	LCAP	4.716	0.546	****	Proteobacteria	Alphaproteobacteria	Caulobacterales	Caulobacteraceae	Caulobacter
ASV142	PHBV	2.871	0.685	**	Proteobacteria	Alphaproteobacteria	Caulobacterales	Caulobacteraceae	Caulobacter
ASV143	PHBV	6.016	0.796	****	Proteobacteria	Gammaproteobacteria	Burkholderiales	Burkholderiaceae	BCP
ASV148	LCAP	4.534	0.698	****	Myxococcota	Polyangia	Polyangiales	Sandaracinaceae	Family_Sandaracinaceae
ASV151	Cellulose	3.691	0.727	***	Acidobacteriota	Acidobacteriae	Acidobacteriales	Acidobacteriaceae	Occallatibacter
ASV151	LCAP	4.306	0.582	****	Acidobacteriota	Acidobacteriae	Acidobacteriales	Acidobacteriaceae	Occallatibacter
ASV151	PHBV	2.896	0.734	*	Acidobacteriota	Acidobacteriae	Acidobacteriales	Acidobacteriaceae	Occallatibacter
ASV156	Cellulose	4.659	0.797	****	Acidobacteriota	Acidobacteriae	Bryobacterales	Bryobacteraceae	Bryobacter
ASV156	LCAP	4.248	0.633	****	Acidobacteriota	Acidobacteriae	Bryobacterales	Bryobacteraceae	Bryobacter

ASV156	PHBV	4.297	0.803	****	Acidobacteriota	Acidobacteriae	Bryobacterales	Bryobacteraceae	Bryobacter
ASV157	LCAP	4.301	0.678	****	Verrucomicrobiota	Chlamydiae	Chlamydiales	cvE6	Family_cvE6
ASV158	Cellulose	3.602	0.691	****	Proteobacteria	Alphaproteobacteria	Micropepsales	Micropepsaceae	Family_Micropepsaceae
ASV158	LCAP	4.300	0.556	****	Proteobacteria	Alphaproteobacteria	Micropepsales	Micropepsaceae	Family_Micropepsaceae
ASV158	PHBV	3.819	0.698	****	Proteobacteria	Alphaproteobacteria	Micropepsales	Micropepsaceae	Family_Micropepsaceae
ASV161	Cellulose	3.022	0.589	****	Acidobacteriota	Acidobacteriae	Acidobacteriales	Acidobacteriaceae	Terracidiphilus
ASV161	LCAP	4.385	0.480	****	Acidobacteriota	Acidobacteriae	Acidobacteriales	Acidobacteriaceae	Terracidiphilus
ASV161	PHBV	2.923	0.595	***	Acidobacteriota	Acidobacteriae	Acidobacteriales	Acidobacteriaceae	Terracidiphilus
ASV167	Cellulose	4.689	0.773	****	Proteobacteria	Gammaproteobacteria	Xanthomonadales	Rhodanobacteraceae	Rudaea
ASV167	LCAP	4.127	0.616	****	Proteobacteria	Gammaproteobacteria	Xanthomonadales	Rhodanobacteraceae	Rudaea
ASV167	PHBV	4.527	0.779	****	Proteobacteria	Gammaproteobacteria	Xanthomonadales	Rhodanobacteraceae	Rudaea
ASV168	LCAP	4.977	0.312	****	Myxococcota	Polyangia	Polyangiales	Polyangiaceae	Pajaroellobacter
ASV173	Cellulose	4.563	0.362	****	Proteobacteria	Gammaproteobacteria	Xanthomonadales	Rhodanobacteraceae	Family_Rhodanobacteraceae
ASV173	LCAP	4.468	0.312	****	Proteobacteria	Gammaproteobacteria	Xanthomonadales	Rhodanobacteraceae	Family_Rhodanobacteraceae
ASV173	PHBV	4.726	0.368	****	Proteobacteria	Gammaproteobacteria	Xanthomonadales	Rhodanobacteraceae	Family_Rhodanobacteraceae
ASV175	LCAP	4.336	0.799	****	Proteobacteria	Alphaproteobacteria	Micropepsales	Micropepsaceae	Family_Micropepsaceae
ASV184	LCAP	3.174	0.669	***	Acidobacteriota	Acidobacteriae	Acidobacteriales	Acidobacteriaceae	Occallatibacter
ASV19	Cellulose	4.751	0.740	****	Proteobacteria	Alphaproteobacteria	Micropepsales	Micropepsaceae	Family_Micropepsaceae
ASV19	LCAP	5.424	0.591	****	Proteobacteria	Alphaproteobacteria	Micropepsales	Micropepsaceae	Family_Micropepsaceae
ASV191	PHBV	4.868	0.179	**	Proteobacteria	Alphaproteobacteria	Rhizobiales	Xanthobacteraceae	Afipia
ASV198	LCAP	3.787	0.849	**	Myxococcota	Polyangia	Polyangiales	BIrii41	Family_BIrii41
ASV212	LCAP	4.228	0.778	****	Proteobacteria	Alphaproteobacteria	Micropepsales	Micropepsaceae	Family_Micropepsaceae
ASV215	LCAP	4.674	0.693	****	Acidobacteriota	Acidobacteriae	Acidobacteriales	Order_Acidobacteriales	Order_Acidobacteriales
ASV220	LCAP	2.954	0.607	***	Acidobacteriota	Acidobacteriae	Acidobacteriales	Acidobacteriaceae	Occallatibacter
ASV222	LCAP	3.285	0.841	*	Acidobacteriota	Acidobacteriae	Acidobacteriales	Order_Acidobacteriales	Order_Acidobacteriales
ASV224	Cellulose	3.826	0.999	*	Proteobacteria	Gammaproteobacteria	Xanthomonadales	Rhodanobacteraceae	Rhodanobacter
ASV226	Cellulose	4.765	0.745	****	Armatimonadota	Fimbriimonadia	Fimbriimonadales	Fimbriimonadaceae	Family_Fimbriimonadaceae
ASV226	LCAP	3.818	0.595	****	Armatimonadota	Fimbriimonadia	Fimbriimonadales	Fimbriimonadaceae	Family_Fimbriimonadaceae

ASV226	PHBV	4.534	0.752	****	Armatimonadota	Fimbriimonadia	Fimbriimonadales	Fimbriimonadaceae	Family_Fimbriimonadaceae
ASV227	LCAP	3.658	0.893	**	Proteobacteria	Gammaproteobacteria	Xanthomonadales	Rhodanobacteraceae	Rhodanobacter
ASV227	PHBV	4.693	0.153	*	Proteobacteria	Gammaproteobacteria	Xanthomonadales	Rhodanobacteraceae	Rhodanobacter
ASV239	LCAP	3.052	0.569	****	Acidobacteriota	Acidobacteriae	Acidobacteriales	Acidobacteriaceae	Occallatibacter
ASV246	LCAP	4.385	0.660	****	Verrucomicrobiota	Chlamydiae	Chlamydiales	cvE6	Family_cvE6
ASV256	LCAP	3.758	0.749	***	Myxococcota	Polyangia	Polyangiales	BIrii41	Family_BIrii41
ASV260	Cellulose	2.821	0.728	*	Proteobacteria	Alphaproteobacteria	Elsterales	Elsteraceae	Family_Elsteraceae
ASV260	LCAP	4.582	0.583	****	Proteobacteria	Alphaproteobacteria	Elsterales	Elsteraceae	Family_Elsteraceae
ASV260	PHBV	3.783	0.734	****	Proteobacteria	Alphaproteobacteria	Elsterales	Elsteraceae	Family_Elsteraceae
ASV264	PHBV	4.317	0.943	*	Proteobacteria	Alphaproteobacteria	Rhizobiales	Xanthobacteraceae	Afipia
ASV266	Cellulose	4.291	1.107	**	WPS-2	Phylum_WPS-2	Phylum_WPS-2	Phylum_WPS-2	Phylum_WPS-2
ASV266	LCAP	4.081	0.741	****	WPS-2	Phylum_WPS-2	Phylum_WPS-2	Phylum_WPS-2	Phylum_WPS-2
ASV27	LCAP	3.482	0.674	****	Acidobacteriota	Acidobacteriae	Acidobacteriales	Acidobacteriaceae	Occallatibacter
ASV283	LCAP	4.162	0.674	****	Proteobacteria	Alphaproteobacteria	Micropepsales	Micropepsaceae	Family_Micropepsaceae
ASV289	LCAP	3.936	0.744	****	Proteobacteria	Alphaproteobacteria	Acetobacterales	Acetobacteraceae	Family_Acetobacteraceae
ASV292	LCAP	4.224	0.656	****	Verrucomicrobiota	Chlamydiae	Chlamydiales	cvE6	Family_cvE6
ASV294	PHBV	4.042	1.030	*	Proteobacteria	Alphaproteobacteria	Rhizobiales	Xanthobacteraceae	Afipia
ASV300	LCAP	3.518	0.898	*	Actinobacteriota	Thermoleophilia	Gaiellales	Order_Gaiellales	Order_Gaiellales
ASV300	PHBV	4.416	1.160	*	Actinobacteriota	Thermoleophilia	Gaiellales	Order_Gaiellales	Order_Gaiellales
ASV305	LCAP	4.152	0.628	****	Acidobacteriota	Acidobacteriae	Acidobacteriales	Acidobacteriaceae	Terracidiphilus
ASV325	LCAP	4.131	0.520	****	Acidobacteriota	Acidobacteriae	Acidobacteriales	Acidobacteriaceae	Terracidiphilus
ASV325	PHBV	2.456	0.649	*	Acidobacteriota	Acidobacteriae	Acidobacteriales	Acidobacteriaceae	Terracidiphilus
ASV33	LCAP	4.859	0.928	****	Verrucomicrobiota	Chlamydiae	Chlamydiales	cvE6	Family_cvE6
ASV340	LCAP	4.038	0.664	****	Proteobacteria	Alphaproteobacteria	Micropepsales	Micropepsaceae	Family_Micropepsaceae
ASV342	LCAP	3.668	0.902	*	Acidobacteriota	Acidobacteriae	Acidobacteriales	Acidobacteriales	Order_Acidobacteriales
ASV343	LCAP	4.023	0.663	****	Proteobacteria	Alphaproteobacteria	Micropepsales	Micropepsaceae	Family_Micropepsaceae
ASV344	LCAP	4.050	0.722	****	Proteobacteria	Alphaproteobacteria	Micropepsales	Micropepsaceae	Family_Micropepsaceae
ASV356	LCAP	4.560	0.263	****	Acidobacteriota	Acidobacteriae	Acidobacteriales	Acidobacteriales	Order_Acidobacteriales

ASV357	LCAP	3.689	0.806	**	Proteobacteria	Alphaproteobacteria	Micropepsales	Micropepsaceae	Family_Micropepsaceae
ASV360	LCAP	3.585	0.892	*	Proteobacteria	Alphaproteobacteria	Micropepsales	Micropepsaceae	Family_Micropepsaceae
ASV361	LCAP	4.040	0.693	****	Proteobacteria	Alphaproteobacteria	Micropepsales	Micropepsaceae	Family_Micropepsaceae
ASV362	PHBV	4.135	0.890	***	Proteobacteria	Gammaproteobacteria	Burkholderiales	Burkholderiaceae	BCP
ASV380	LCAP	3.599	0.796	**	Myxococcota	Polyangia	Polyangiales	Sandaracinaceae	Family_Sandaracinaceae
ASV404	LCAP	3.941	0.713	****	Proteobacteria	Alphaproteobacteria	Micropepsales	Micropepsaceae	Family_Micropepsaceae
ASV420	PHBV	5.338	0.630	****	Proteobacteria	Gammaproteobacteria	Burkholderiales	Burkholderiaceae	BCP
ASV423	LCAP	3.592	0.789	**	Myxococcota	Polyangia	Polyangiales	BIrii41	Family_BIrii41
ASV430	LCAP	3.705	0.697	****	Myxococcota	Polyangia	Polyangiales	Sandaracinaceae	Family_Sandaracinaceae
ASV448	LCAP	3.443	0.864	*	Myxococcota	Polyangia	Polyangiales	BIrii41	Family_BIrii41
ASV457	PHBV	4.141	0.851	***	Proteobacteria	Gammaproteobacteria	Burkholderiales	Burkholderiaceae	BCP
ASV468	PHBV	4.454	0.794	****	Proteobacteria	Gammaproteobacteria	Burkholderiales	Burkholderiaceae	BCP
ASV469	PHBV	5.274	0.551	****	Proteobacteria	Gammaproteobacteria	Burkholderiales	Burkholderiaceae	BCP
ASV479	PHBV	5.215	0.611	****	Proteobacteria	Gammaproteobacteria	Burkholderiales	Burkholderiaceae	BCP
ASV51	LCAP	2.665	0.722	*	Acidobacteriota	Acidobacteriae	Acidobacteriales	Order_Acidobacteriales	Order_Acidobacteriales
ASV527	Cellulose	4.563	0.381	****	Planctomycetota	Phycisphaerae	Tepidisphaerales	WD2101 soil group	Family_WD2101 soil group
ASV527	PHBV	4.584	0.387	****	Planctomycetota	Phycisphaerae	Tepidisphaerales	WD2101 soil group	Family_WD2101 soil group
ASV540	LCAP	3.693	0.636	****	Proteobacteria	Alphaproteobacteria	Micropepsales	Micropepsaceae	Family_Micropepsaceae
ASV596	LCAP	3.433	0.808	**	Planctomycetota	Planctomycetes	Gemmatales	Gemmataceae	Gemmata
ASV601	LCAP	3.371	0.752	**	Proteobacteria	Alphaproteobacteria	Micropepsales	Micropepsaceae	Family_Micropepsaceae
ASV605	PHBV	4.629	0.903	****	Proteobacteria	Gammaproteobacteria	Burkholderiales	Burkholderiaceae	BCP
ASV652	PHBV	2.968	0.719	**	Proteobacteria	Alphaproteobacteria	Rhizobiales	Xanthobacteraceae	Family_Xanthobacteraceae
ASV654	LCAP	3.336	0.739	**	Proteobacteria	Gammaproteobacteria	KF-JG30-C25	Order_KF-JG30-C25	Order_KF-JG30-C25
ASV675	Cellulose	4.634	1.075	**	Acidobacteriota	Holophagae	Subgroup 7	Order_Subgroup 7	Order_Subgroup 7
ASV688	PHBV	5.021	0.352	****	Proteobacteria	Gammaproteobacteria	Burkholderiales	Burkholderiaceae	BCP
ASV77	LCAP	4.727	0.818	****	Proteobacteria	Alphaproteobacteria	Micropepsales	Micropepsaceae	Family_Micropepsaceae
ASV85	LCAP	4.130	0.638	****	Acidobacteriota	Acidobacteriae	Acidobacteriales	Acidobacteriaceae	Terracidiphilus
ASV89	Cellulose	4.444	0.731	****	Acidobacteriota	Acidobacteriae	Acidobacteriales	Koribacteraceae	Candidatus Koribacter

ASV89	LCAP	3.946	0.585	****	Acidobacteriota	Acidobacteriae	Acidobacteriales	Koribacteraceae	Candidatus Koribacter
ASV89	PHBV	3.974	0.737	****	Acidobacteriota	Acidobacteriae	Acidobacteriales	Koribacteraceae	Candidatus Koribacter
ASV9	PHBV	2.596	0.469	****	Proteobacteria	Gammaproteobacteria	Burkholderiales	Burkholderiaceae	BCP

D. Supplementary References

1. Eck, M. Degradable polyethylene-like materials. PhD thesis, Universität Konstanz, 2023.2. Lott C et al. Field and mesocosm methods to test biodegradable plastic film under marine conditions. *PLoS One* 2020;**15**:e0236579. https://doi.org/10.1371/journal.pone.0236579

3. Avasthi I et al. Biodegradable mineral plastics. *Small Methods* 2024;**8**:2300575. https://doi.org/10.1002/smtd.202300575

4. Lerner H et al. Unkaputtbar – oder nicht? *Nachr Chem* 2025;**73**:60–62. https://doi.org/10.1002/nadc.20254149008

5. Larsen MH et al. Genetic manipulation of *Mycobacterium tuberculosis*. *Curr Protoc Microbiol* 2007;**6**:10A.2.1-10A.2.21. https://doi.org/10.1002/9780471729259.mc10a02s6

6. Berthelet M, Whyte LG, Greer CW. Rapid, direct extraction of DNA from soils for PCR analysis using polyvinylpolypyrrolidone spin columns. *FEMS Microbiol Lett* 1996;**138**:17–22. https://doi.org/10.1111/j.1574-6968.1996.tb08128.x

7. Klindworth A et al. Evaluation of general 16S ribosomal RNA gene PCR primers for classical and next-generation sequencing-based diversity studies. *Nucleic Acids Res* 2013;**41**:1–11. https://doi.org/10.1093/nar/gks808

8. Purahong W et al. Back to the future: decomposability of a biobased and biodegradable plastic in field soil environments and Its microbiome under ambient and future climates. *Environ Sci Technol* 2021;**55**:12337–12351. https://doi.org/10.1021/acs.est.1c02695

9. Lerner H et al. Culture-independent analysis of linuron-mineralizing microbiota and functions in on-farm biopurification systems via DNA-Stable Isotope Probing: comparison with enrichment culture. *Environ Sci Technol* 2020;**54**:9387–9397. https://doi.org/10.1021/acs.est.0c02124

10. Quast C et al. The SILVA ribosomal RNA gene database project: Improved data processing and web-based tools. *Nucleic Acids Res* 2013;**41**:590–596. https://doi.org/10.1093/nar/gks1219

11. Abarenkov K et al. The UNITE database for molecular identification and taxonomic communication of fungi and other eukaryotes: sequences, taxa and classifications reconsidered. *Nucleic Acids Res* 2024;**52**:D791–D797. https://doi.org/10.1093/nar/gkad1039

12. McMurdie PJ, Holmes S. phyloseq: an R package for reproducible interactive analysis and graphics of microbiome census data. *PLoS One* 2013;**8**:e61217. https://doi.org/10.1371/journal.pone.0061217

13. Pagès, H. et al. Biostrings: Efficient manipulation of biological strings. R package (2019).

14. Paradis E, Schliep K. ape 5.0: an environment for modern phylogenetics and evolutionary analyses in R. *Bioinformatics* 2019;**35**:526–528. https://doi.org/10.1093/bioinformatics/bty633

15. Oksanen J et al. vegan: Community Ecology Package. *CRAN: Contributed Packages* (2001).

16. Lin H, Peddada S Das. Analysis of compositions of microbiomes with bias correction. *Nat Commun* 2020;**11**:3514. https://doi.org/10.1038/s41467-020-17041-7

17. Wickham H. ggplot2: elegant graphics for data analysis. New York: Springer-verlag, 2016.

18. Eren AM et al. A filtering method to generate high quality short reads using Illumina paired-end technology. *PLoS One* 2013;**8**:e66643. https://doi.org/10.1371/journal.pone.0066643

19. Eren AM et al. Community-led, integrated, reproducible multi-omics with anvi'o. *Nat Microbiol* 2020;**6**:3–6. https://doi.org/10.1038/s41564-020-00834-3

20. Langmead B, Salzberg SL. Fast gapped-read alignment with Bowtie 2. *Nat Methods* 2012;**9**:357–359. https://doi.org/10.1038/nmeth.1923

21. Hyatt D et al. Prodigal: prokaryotic gene recognition and translation initiation site identification. *BMC Bioinformatics* 2010;**11**:119. https://doi.org/10.1186/1471-2105-11-119

22. Parks DH et al. GTDB: an ongoing census of bacterial and archaeal diversity through a phylogenetically consistent, rank normalized and complete genome-based taxonomy. *Nucleic Acids Res* 2022;**50**:D785–D794. https://doi.org/10.1093/nar/gkab776

23. Danso D et al. New insights into the function and global distribution of polyethylene terephthalate (PET)-degrading bacteria and enzymes in marine and terrestrial metagenomes. *Appl Environ Microbiol* 2018;**84**. https://doi.org/10.1128/AEM.02773-17

24. Gambarini V et al. PlasticDB: a database of microorganisms and proteins linked to plastic biodegradation. *Database* 2022;**2022**:baac008. https://doi.org/10.1093/database/baac008

25. Katoh K, Standley DM. MAFFT multiple sequence alignment software version 7: improvements in performance and usability. *Mol Biol Evol* 2013;**30**:772–780. https://doi.org/10.1093/molbev/mst010

26. Eddy SR. Accelerated profile HMM searches. *PLoS Comput Biol* 2011;**7**:e1002195. https://doi.org/10.1371/journal.pcbi.1002195

27. Camacho C et al. BLAST+: architecture and applications. *BMC Bioinformatics* 2009;**10**:421. https://doi.org/10.1186/1471-2105-10-421

28. Teufel F et al. SignalP 6.0 predicts all five types of signal peptides using protein language models. *Nat Biotechnol* 2022;**40**:1023–1025. https://doi.org/10.1038/s41587-021-01156-3

29. Lê S, Josse J, Husson F. FactoMineR : an R package for multivariate analysis. *J Stat Softw* 2008;**25**:1–18. https://doi.org/10.18637/jss.v025.i01

30. Abramson J et al. Accurate structure prediction of biomolecular interactions with AlphaFold 3. *Nature* 2024;**630**:493–500. https://doi.org/10.1038/s41586-024-07487-w

31. Laskowski RA, Watson JD, Thornton JM. ProFunc: a server for predicting protein function from 3D structure. *Nucleic Acids Res* 2005;**33**:W89–W93. https://doi.org/10.1093/nar/gki414

32. Weininger D. SMILES, a chemical language and information system. 1. Introduction to methodology and encoding rules. *J Chem Inf Comput Sci* 1988;**28**:31–36. https://doi.org/10.1021/ci00057a005

33. O'Boyle NM et al. Open Babel: an open chemical toolbox. *J Cheminform* 2011;**3**:33. https://doi.org/10.1186/1758-2946-3-33

34. Morris GM et al. AutoDock4 and AutoDockTools4: automated docking with selective receptor flexibility. *J Comput Chem* 2009;**30**:2785–2791. https://doi.org/10.1002/jcc.21256

35. Goddard TD et al. UCSF ChimeraX: meeting modern challenges in visualization and analysis. *Protein Science* 2018;**27**:14–25. https://doi.org/10.1002/pro.3235

36. Katoh K, Rozewicki J, Yamada KD. MAFFT online service: multiple sequence alignment, interactive sequence choice and visualization. *Brief Bioinform* 2019;**20**:1160–1166. https://doi.org/10.1093/bib/bbx108

37. Hunter JD. Matplotlib: a 2D graphics environment. *Comput Sci Eng* 2007;**9**:90–95. https://doi.org/10.1109/MCSE.2007.55

38. Cock PJA et al. Biopython: freely available Python tools for computational molecular biology and bioinformatics. *Bioinformatics* 2009;**25**:1422–1423. https://doi.org/10.1093/bioinformatics/btp163

39. Novy R, Morris B. Use of glucose to control basal expression in the pET system. *Innovations* 2001;**13**:13–15.

40. Peng Y et al. Accurately determining esterase activity via the isosbestic point of p-nitrophenol. *Bioresources* 2016;**11**. https://doi.org/10.15376/biores.11.4.10099-10111

41. Yoshida S et al. A bacterium that degrades and assimilates poly(ethylene terephthalate). *Science* 2016;**351**:1196–9. https://doi.org/10.1126/science.aad6359

42. Eck M et al. Biodegradable high-density polyethylene-like material. *Angewandte Chemie International Edition* 2023;**62**:e202213438. https://doi.org/https://doi.org/10.1002/anie.202213438
